# Physical Factors Correlate to Microbial Community Structure and Nitrogen Cycling Gene Abundance in a Nitrate Fed Eutrophic Lagoon

**DOI:** 10.3389/fmicb.2016.01691

**Published:** 2016-10-25

**Authors:** Matthew P. Highton, Stéphanie Roosa, Josie Crawshaw, Marc Schallenberg, Sergio E. Morales

**Affiliations:** ^1^Department of Microbiology and Immunology, Otago School of Medical Sciences, University of OtagoDunedin, New Zealand; ^2^Department of Marine Science, University of OtagoDunedin, New Zealand; ^3^Department of Zoology, University of OtagoDunedin, New Zealand

**Keywords:** qPCR, sediment grain size, denitrification, 16S rRNA, nutrient, ICOLL, DEA

## Abstract

Nitrogenous run-off from farmed pastures contributes to the eutrophication of Lake Ellesmere, a large shallow lagoon/lake on the east coast of New Zealand. Tributaries periodically deliver high loads of nitrate to the lake which likely affect microbial communities therein. We hypothesized that a nutrient gradient would form from the potential sources (tributaries) creating a disturbance resulting in changes in microbial community structure. To test this we first determined the existence of such a gradient but found only a weak nitrogen (TN) and phosphorous gradient (DRP). Changes in microbial communities were determined by measuring functional potential (quantification of nitrogen cycling genes via *nifH*, *nirS*, *nosZI*, and *nosZII* using qPCR), potential activity (via denitrification enzyme activity), as well as using changes in total community (via 16S rRNA gene amplicon sequencing). Our results demonstrated that changes in microbial communities at a phylogenetic (relative abundance) and functional level (proportion of the microbial community carrying *nifH* and *nosZI* genes) were most strongly associated with physical gradients (e.g., lake depth, sediment grain size, sediment porosity) and not nutrient concentrations. Low nitrate influx at the time of sampling is proposed as a factor contributing to the observed patterns.

## Introduction

Conventional agricultural practices result in large inputs of nutrients into soils either to promote crop and livestock growth or as waste products. Not all of these nutrients can be consumed locally, inevitably leaving excess which affects local and distant environments (Tilman, 1999; Di and Cameron, 2002). Through runoff or seepage to groundwater, nutrients can reach aquatic ecosystems ranging from freshwater to salt water (Tilman, 1999; Di and Cameron, 2002; Camargo and Alonso, 2006). Agriculture is a cornerstone of the New Zealand economy and accounts for much of its land use practices (Statistics New Zealand, 2012). Thus, New Zealand is an ideal model for studying the environmental impacts of agriculture. In particular, nitrogen wastes are of concern as they can increase greenhouse gas emissions through N_2_O production (Canfield et al., 2010), and can lead to eutrophication of aquatic environments likely imposing a selective pressure than can disturb intrinsic community assembly processes.

Lake Ellesmere/Te Waihora is a large coastal lagoon located on the east coast of New Zealand’s South Island and has been described as a hyper-eutrophic lake (Hughey et al., 2013). The lagoon is intermittently connected to the ocean [such systems have been previously described as Intermittently Closed and Open Lakes and Lagoons or ICOLLs (Roy et al., 2001)], while also receiving inflows from nutrient rich freshwater sources (streams, groundwater) draining a farmed catchment. These factors contribute to large temporal variation in water salinity, nutrient levels, phytoplankton biomass (Schallenberg et al., 2010) and probably also microbial communities (Scofield et al., 2015). Similar lagoon systems act as net sinks of land derived nitrogen (N) and phosphorus (P) (Kjerfve, 1994), but diverse outcomes for deposited N are possible upon eutrophication (Taylor et al., 1996; Howarth and Marino, 2006; Nixon and Fulweiler, 2009; Glibert et al., 2014). Surprisingly, Lake Ellesmere represents an example where, despite high nitrogen loading rates to the lake, on average, lake water has approximately 1.5-fold lower Total Nitrogen concentration (TN) than inflowing tributaries (Schallenberg et al., 2010) and 10-fold lower Total Nitrogen-to-Phosphorus ratio (TN:TP) (Schallenberg et al., 2010). This relative N deficit could be accounted for by microbial processes such as denitrification and anaerobic ammonium oxidation (anammox) (Zhu et al., 2013). Denitrification (Knowles, 1982), the stepwise reduction of nitrate to N_2_ gas, has been suggested as a potential pathway for the removal of N from the lake (Larned and Schallenberg, 2006). Irrespective of the N removal pathway, the episodic inflow of high nutrients from farming is predicted to disturb natural processes, imposing an alternative selective pressure (i.e., disturbance). This high nutrient inflow into lake Ellesmere may affect benthic microbes involved in nutrient cycling (like denitrifiers) as well as other members of the community. These linked changes in functional potential and total microbial community structure have been demonstrated before with decreases in microbial richness resulting in a reduction of the denitrification activity in soils (Philippot et al., 2013), while community composition as determined via 16S rRNA gene can be linked to denitrification outcomes in an environment (Morales et al., 2015). Within estuaries both salinity (Campbell and Kirchman, 2013) and nutrient gradients (Smith et al., 2015) have been shown to influence community composition, and changes in microbial communities have been suggested as bioindicators of terrestrial inputs (Li et al., 2013). Furthermore, the benthic microbial communities of ICOLLS and Lake Ellesmere in particular remain poorly described, even though the large surface area relative to lagoon volume should make them a relatively important component of lake functioning.

Here, we carried out physicochemical profiling of 18 lake sites along with concomitant measurement of genetic (qPCR of denitrifying genes *nirS*, *nosZI*, *nosZII*), enzymatic denitrification potential (denitrification enzyme assay) and high throughput sequencing of the 16S rRNA gene. We aimed to determine: (i) whether nutrient inputs from tributaries, or groundwater, were impacting the nutrient status of the lake resulting in a gradient from the two predicted main sources of nutrients, and (ii) the effect of these inflows on microbial communities at both a total community (16S) and targeted (functional) level within lake sediments. It was hypothesized that nitrate rich inflows from the lagoon’s main tributaries would form a nutrient gradient across the lake, creating a selective force (disturbance) resulting in changes at both functional (N cycling genes and enzymatic potential) and overall microbial community composition.

## Materials and Methods

### Site Description

**FIGURE 1:**
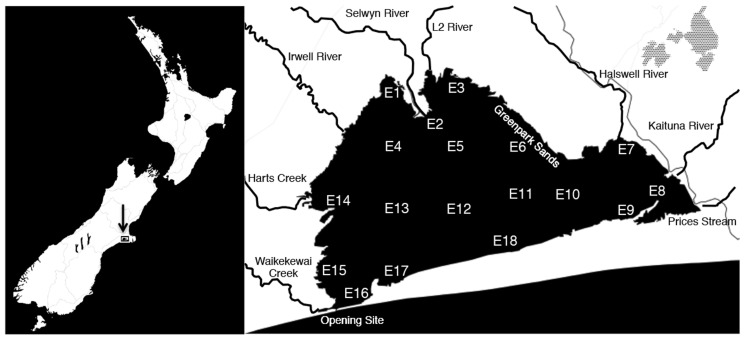
**Map of Lake Ellesmere and location of all 18 sampling sites.** Arrow indicates location of Lake Ellesmere on New Zealand’s East coast (left panel). Major tributaries and features are labeled on right panel.

Lake Ellesmere/Te Waihora is a large (16,000 ha) shallow (average depth = 1.5 m), intermittently closed and open lake/lagoon (ICOLL) located south of Banks Peninsula on the East Coast of New Zealand’s South Island (Jellyman et al., 2011). The lake receives nitrogen and phosphorus-rich freshwater from a number of rivers and streams including Hart’s Creek and the L2, Selwyn, Irwell, Halswell and Kaituna Rivers (**Figure 1**). A gravel bar located on the southwest edge of the lake is periodically mechanically opened to reduce lake water levels, resulting in water exchange with the ocean (Schallenberg et al., 2010).

### Sediment Sampling

Sampling at 18 lake sites (**Figure 1**; Supplementary Table S1) was carried out on April 9, 10, and 11, 2014. Sediment samples (four replicate sediment samples per site) were collected using a gravity corer with a 75 mm diameter acrylic core tube. The top 4 cm of the sediment column was collected and homogenized before a 2 ml subsample of homogenized sediment was stored in a 2 ml microcentrifuge tube. Replicates were processed and stored separately without compositing. Sediment samples were frozen on dry ice in the field and subsequently stored at -80°C in the lab until processed for microbial community nucleic acid extraction. Bulk sediment oxidation-reduction potential (Eh) was measured from the last homogenized sediment core from each site using a Schott millivolt meter with a redox probe.

Three additional cores were obtained from each site and subsequently pooled. These were used for analyses of sediment particle size, organic matter, porosity and denitrification enzyme activity (DEA) assays. Samples for sediment characteristics were stored on ice in the field, and then frozen at -20°C until analyzed. Samples for DEA were stored on ice in the field, and transported via overnight courier on ice to the Environmental Research Institute, University of Waikato, Hamilton for analysis.

### Water Sampling

Water clarity was measured during a single sampling event at each site using a Secchi disk. Water samples were collected afterwards using a messenger activated van Dorn water sampler submerged to just above the lake bed at each site. One 50 ml water sample was collected and transferred into an acid washed 50 ml tube as dilution water for sediment DEA assays, and two additional 50 ml samples were collected for water nutrient analysis of dissolved inorganic and total N and P concentrations. Samples for dissolved nutrients were filtered through an Advantec GF-75 25 mm glass fiber filter (nominal pore size = 0.7 μm) into a 50 ml acid washed tube. All samples were stored on ice in the field and were frozen at -20°C upon returning to the lab. Physicochemical variables (temperature, salinity, and conductivity) were measured using an YSI Professional Plus multiparameter meter (YSI Environmental, Yellow Springs, OH, USA).

### Water and Sediment Analyses

The sum of nitrate and nitrite (NO_2_^-^+NO_3_^-^), dissolved reactive phosphorus (DRP), and the sum of ammonia and ammonium, (NH_3_ and NH_4_^+^) were measured from GF-75-filtered water samples using a SANPlus segmented flow colorimetric autoanalyzer (SkalarAnalytical B.V., Breda, The Netherlands), as described previously (Schallenberg and Burns, 2004). Total nitrogen (TN) and total phosphorus (TP) were measured on unfiltered water samples as described above, but after wet oxidation at 121°C. Sediment organic matter content (organic matter %) was measured as gravimetric loss on ignition at 450°C, as a proportion of the sediment dry weight. Sediment porosity was calculated as the percentage of sediment wet weight contributed by water (dried to a constant mass at 60°C). Sediment fractions attributable to sand (63–2000 μm), silt (2–63 μm), and clay (0–2 μm) were determined using a Mastersizer 2000 laser diffraction particle size analyzer (Malvern Instruments, Malvern, Worcestershire, UK) after sediments had been pre-treated with hydrogen peroxide to remove organic matter.

### Denitrification Enzyme Assay

Denitrification enzyme activity was measured at the Environmental Research Institute (University of Waikato, Hamilton) by an acetylene block assay on diluted lake sediments using the method of Bruesewitz et al. (2011). Briefly, four different treatments (control, 10 mg/L nitrate, 12 mg/L glucose, 10 mg/L nitrate plus 12 mg/L glucose) were applied to homogenized lake water (15 ml) and sediments (15 ml) from the 18 Ellesmere sites in the presence of acetylene, to block the conversion of N_2_O to N_2_. Sediment-water mixes were incubated at room temperature (22°C) in 45 ml glass bottles with gas tight silicone septa. Gas samples (8 ml) were taken every hour, over 6 h, and measured using a Varian CP 3800 gas chromatograph with an ECD detector. When denitrification progressed linearly the formulas (1) and (2) were used to calculate the rate of denitrification where P is the amount of N_2_O produced at time t, q is the DEA and N_0_ is the number of bacteria at time 0.

(1) $$P=P_{0}+qN_{0}t$$

(2) $$\frac{dp}{dt}=qN_{0}$$

When denitrification progressed exponentially the formulas (3) and (4) were used where μ is the specific growth rate constant.

(3) $$P=P_{0}+\frac{qN_{0}}{\mu }\left(e^{\mu t}-1\right)$$

(4) $$\frac{dp}{dt}=qN_{0}e^{\mu t}$$

### DNA Extraction

Total microbial community DNA was extracted from all 72 samples (18 sites, 4 replicates) following a modified Griffiths et al. (2000) protocol (Paulin et al., 2013). Briefly, extractions were performed with 1 g 0.5 mm silica beads, 0.75 g of 0.1 mm silica beads, 0.5 g of sediment (wet weight), low-molecular weight salmon sperm DNA (500 mg/L final concentration; Invitrogen, Carlsbad, CA, USA), 500 μL cetyltrimethyl ammonium bromide (CTAB) buffer and 500 μl phenol-chloroform-isoamyl alcohol 25:24:1 (PCI, ACROS Organics, Geel, Belgium). Samples were lysed in a Genogrinder (SPEX CertiPrep, Metuchen, NJ, USA) using two 15-s intervals of bead beating at 1750 rpm with an intermittent cooling on ice. The aqueous phase containing nucleic acids was separated by centrifugation at 10,000 rpm for 10 min (4°C) and a subsequent purification with PCI was performed. Nucleic acids were then precipitated by a 2 h incubation on ice using 20% final concentration polyethylene glycol 6000 (PEG). Following centrifugation (13,000 rpm, 10 min, 4°C) the pellet was washed with 70% ice-cold ethanol, air dried and then resuspended in 50 μL of sterile water. A 40 μL volume of co-extract was then incubated at 37°C with 4 μL of 20 Unit/μL RNase I (Ambion, Austin, TX, USA) for 30 min in a 50 μL solution (final volume) of 0.2 M NaCl to degrade co-extracted RNA. DNA was quantified and assessed for purity as well as humics contamination using a Nanodrop 1000 (ThermoScientific, Wilmington DE, USA). DNA was stored at -20°C until downstream analyses.

### Quantitative PCR (qPCR)

Quantitative PCR targeting two functional groups controlling net outflow and inflow of nitrogen denitrification [*nirS* (Throback et al., 2004), cytochrome cd1 nitrite reductase; *nosZ*I (Henry et al., 2006) and *nosZ*II (Jones et al., 2013), Clades I and II nitrous oxide reductase, respectively] and nitrogen fixation [*nifH*, nitrogenase (Rösch and Bothe, 2005)], as well as total microbial abundance (16S rRNA gene; Hartman et al., 2009) was carried out for all samples using the primer pairs and thermocycling conditions described in **Table 1**. For each DNA sample, at least triplicate 10 μL (technical replicates) reactions were plated into 384 well plates (Applied Biosystems) using an automated VERSA liquid handling robot (Aurora, Vancouver, B.C., Canada). Reactions contained 10 ng of DNA, 5 μL of Master Mix and 0.5 μM of each primer excluding *nosZ*II reactions that contained 1 μM of each primer. All *nifH*, *nirS*, *nosZ*I, 16S amplifications were performed using Fast SYBR Green Master Mix (Applied Biosystems, Foster City, CA, USA) and all *nosZ*II amplifications were performed using Luminaris Color HiGreen low ROX Master Mix (ThermoScientific). pGEM-T easy (Promega, Madison, WI, USA) cloned template standards were included in every run to allow absolute quantification of templates. Amplification was performed on the ViiA 7 real time qPCR machine (Applied Biosystems). Melt curves were produced with each run to confirm amplification of the desired gene target and absence of contamination on negative controls.

**Table 1:** Quantitative PCR (qPCR) primers and conditions used for the amplifications of *nosZI*, *nosZII*, *nirS*, *nifH*, 16SrRNA gene.

Target	Forward primer	Reverse primer	Primer reference	Hold stage	PCR stage	Complete extension	Melt curve
*nosZII*	1153_nosZ8F- CTIGGICCIY TKCAYAC	1888_nosZ29R- GCIGAICARA AITCBGTRC	Jones et al., 2013	50°C, 95°C	95°C, **60–54°C**, 72°C, 80°C	-	95°C, 60°C, 95°C, 60°C
				2 min, 10 min	15 s, 30 s, 30 s, 30 s	-	15 s, 1 min, 30 s, 15 s
					-44 cycles at 54°C annealing temperature		
*nifH*	nifHF-AAAGGYGGWATCGG YAARTCCACCAC	nifHRb-TGSGCYTTGTCYTCR CGGATBGGCAT	Rösch and Bothe, 2005	96°C	96°C, **58.5°C**, 68°C	72°C	96°C, 50°C, 96°C, 72°C
				10 min	3 s, 3 s, 30 s	5 min	15 s,1 min, 15 s, 5 min
					-40cycles		
*nosZI*	nosZIF-CGCRACGGCAAS AAGGTSMSSGT	nosZIR-CAKRTGCAKSGC RTGGCAGAA	Henry et al., 2006	96°C	96°C, **58.5°C**, 68°C	72°C	96°C, 50°C, 96°C
				10 min	3 s, 3 s, 30 s	5 min	15 s, 1 min, 15 s


					-40 cycles		
*nirS*	cd3aF-GTSAACGTSAA GGARACSGG	R3cd-GASTTCGGRTGS GTCTTGA	Throback et al., 2004	95°C	95°C, **58.5°C**, 72°C, 77°C	72 C	95°C, 60°C, 95°C
				10 min	10 s, 20 s, 20 s, 20 s -40 cycles	5 min	15 s, 1 min, 15 s
16S rRNA gene	16S UNIV-F-ACT CCT ACG GGA GGC AGC AG	16S UNIV-R-ATT ACC GCG GCT GCT GGC	Hartman et al., 2009	95°C	95°C, **65°C**, 72°C	-	95°C, 60°C, 95°C, 72°C
				15 s	15 s, 20 s, 20 s -40 cycles	-	15 s, 1 min, 15 s, 5 min

*Annealing temperatures are in bold*.

### 16S rRNA Gene Amplicon Sequencing and Data Processing

Gene amplification and sequencing were performed using Version 4_13 of the Earth Microbiome Project standard protocol (Caporaso et al., 2012) targeting the 515f-806r region of the 16S rRNA gene. All samples were loaded on a single Illumina MiSeq run. Raw sequences were quality filtered using Qiime 1.9.0 default parameters (Caporaso et al., 2010) before processing using an open reference Operational Taxonomic Unit (OTU) picking strategy using the SILVA version 119 reference library (Quast et al., 2013) and UCLUST (Edgar, 2010). All sequences were binned into clusters based on 97% sequence similarity. Taxonomy was assigned using the SILVA database and BLAST (Altschul et al., 1990). The sequence pool was then randomly subsampled 10 times to a depth of 17400 sequences using the multiple_rarefactions_even_depth.py script to eliminate biases in the depth of sampling. Resulting OTU tables were merged before further analysis.

### Statistical and Microbial Community Analyses

Downstream 16S analyses were carried out in Phyloseq (McMurdie and Holmes, 2013) for R (R Development Core Team, 2008) using a QIIME generated biom file and lake metadata. The ordination of lake sites was calculated using the relative abundance of OTUs. A Bray–Curtis dissimilarity matrix was used when appropriate. Direct and indirect gradient ordination methods were used to scrutinize the overall trends in microbial community data. Spearman’s correlations, principal component analysis (PCA) and linear regression of lake metadata were performed using JMP 11.2 (SAS institute. Cary, NC, USA). Spatial contour plots were produced in Surfer 11 (Golden software, Golden, CO, USA) using the kriging interpolation algorithm. Construction of a Spearmans network was carried out using qgraph (Epskamp et al., 2012).

### Accession Numbers

All 16S rRNA gene sequences have been submitted to NCBI under accession numbers SRP068133.

## Results

### Physical and Chemical Gradients

**FIGURE 2:**
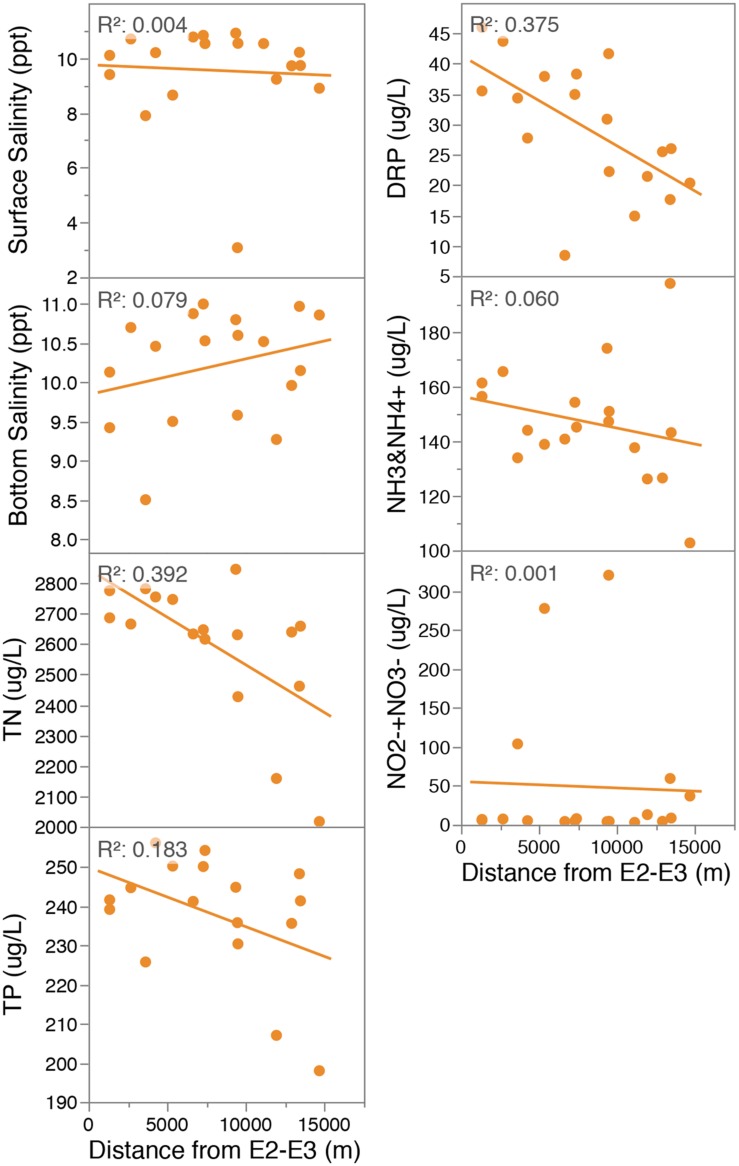
**Linear regression of water chemistry data (18 points, 1 measurement per site) against distance from E2-3 (midpoint between the Selwyn and L2 Rivers [the expected major inflows of freshwater and nutrients]) (right column).** All *p* values > 0.05 with the exceptions of DRP and TN which were < 0.01). Site E17 was excluded as an outlier from linear regression plots for variables TN and TP. TN: Total Nitrogen; TP: Total Phosphorus; DRP: Dissolved Reactive Phosphorus; NH_3_ and NH_4_^+^: sum of ammonia and ammonium; NO_2_^-^+NO_3_^-^: sum of nitrite and nitrate.

**FIGURE 3:**
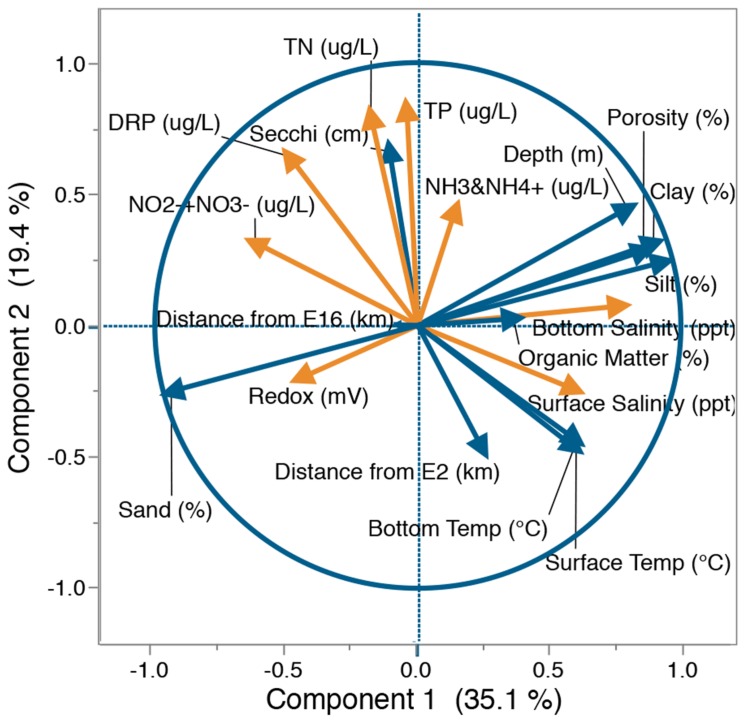
**Principal component analysis (PCA) of physical (blue) and chemical (orange) parameters within Lake Ellesmere.** Factor loadings based on separation of 18 lake sites by water chemistry (TN, TP, NH_3_ and NH_4_^+^, NO_2_^-^+NO_3_^-^, DRP, surface salinity, bottom salinity), sediment chemistry (organic matter%, redox potential) and physical factors (depth, sand%, silt%, clay%, Secchi, porosity, bottom temperature, distance from site E2, distance from site E16). Axis percentage values indicate % of variability explained by that axis. TN, Total Nitrogen concentration; TP, Total Phosphorus concentration; NH_3_ and NH_4_^+^, sum of ammonia and ammonium; NO_2_^-^+NO_3_^-^, sum of nitrite and nitrate; DRP, Dissolved Reactive Phosphorus; Secchi: Secchi depth.

Physical and chemical gradients within Lake Ellesmere’s sediment and water column were analyzed across 18 sites (**Figures 1**–**3**; Supplementary Figures S1–S6). To determine the influence of the main freshwater source (and largest predicted source of incoming nutrients) chemical values were correlated to the distance from the northern boundary of the lake at the inflow of the Selwyn and L2 rivers (sites E2 and E3). These two sites contribute the largest yearly bulk water inflow into the lake (3.77 and 2.3 m^3^s^-1^ mean flow, 2009–2014). TN and DRP had weak but significant (*p* < 0.01) negative correlations with distance from the inflow of the Selwyn and L2 Rivers (midpoint between sites E2 and E3) and decreased 1.4- and 2.3-fold, respectively, from E2 to the most distant site (E8) (**Figure 2**). In an alternative measure, Lake salinity was used as a predictor for freshwater inflow, and correlated to nutrient concentrations within the lake waters to determine the influence of freshwater inflow on water column nutrient status. Lower surface and bottom-water salinity was observed at sites along the north, west and eastern edges of the lake suggesting influence from freshwater rivers and possibly groundwater (Supplementary Figure S1). Surface water salinity was significantly correlated to NO_2_^-^+NO_3_^-^ and NH_3_ and NH_4_^+^ as determined by Spearman’s rank correlation [Supplementary Table S2, Spearmans rho (ρ) = -0.7548, 0.5225]. However, in linear regression the relationship with NO_2_^-^+NO_3_^-^ was heavily weighted by Site E14 (at the inflow of Hart’s Creek), which had a high NO_2_^-^+NO_3_^-^ (320 μg/L) concentration and low surface salinity (3.08 ppt) (Supplementary Figure S2). Removal of this data point reduces significance and the coefficient of determination to 0.0102 and 0.37, respectively.

Principal component analysis of physical and chemical data was used to identify correlations between measured lake variables and the major gradients across the lake. Factor loadings demonstrated that salinity and sediment physical characteristics tended to co-segregate with the first principal component (PC1) explaining 35.1% of the variability between sites (**Figure 3**). Further analysis by Spearman’s rank correlation (Supplementary Table S2) and visual inspection of lake parameter spatial distributions (Supplementary Figure S3) confirmed an association between these parameters; through the deeper central region and south eastern perimeter of the lake, sediments were predominantly composed of silt and clay with higher levels of porosity and organic matter, while the northern perimeter was dominated by sand. Characteristics of the lake water were mainly associated with the second component (PC2), accounting for 19.4% of the variability (**Figure 3**). Spearman’s rank correlation (Supplementary Table S2) and visual analysis of lake parameter spatial distributions (Supplementary Figure S1) confirmed that unlike physical factors, chemical parameters showed no common distribution. For a full description of lake chemical and physical parameter distributions see Supplementary Text 1.

### Denitrification Enzyme Activity

**FIGURE 4:**
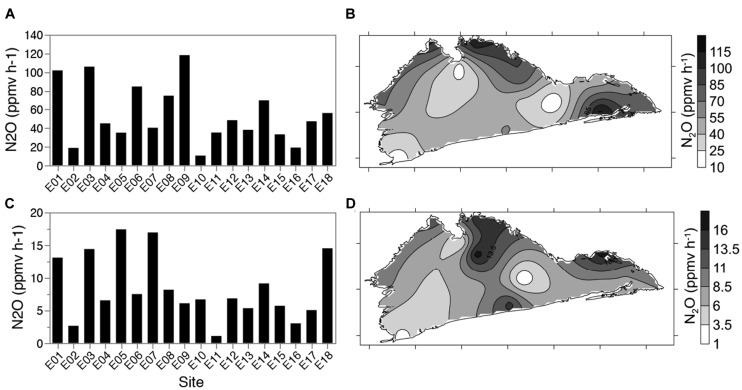
**Denitrification enzyme activity (DEA; N_2_O production h^-1^) over 18 lake sites.** Sediment samples were amended with nitrate + carbon (glucose) **(A,B)** or nitrate only **(C,D)** and N_2_O emissions measured over 6 h. Map figures **(B)** and **(D)** represent 18 data points interpolated by kriging.

Denitrification enzyme activity was used to determine changes in enzymatic potential across the lake, and in response to any physicochemical gradients suggesting changes in microbial communities. DEA was highest for sediments amended with both carbon and nitrogen (DEA +C+N) (55 ± 31 ppmv of N_2_O h^-1^) (**Figures 4A,B**). Nitrate only (DEA +N) amended sediments showed a similar pattern of activity (8 ± 5 ppmv of N_2_O h^-1^) but with a 6.5-fold lower average N_2_O production, ranging from 1.1 ppmv of N_2_O h^-1^ at site E11 and 17.5 ppmv of N_2_O h^-1^ at site E5 (**Figures 4C,D**). Assays amended with carbon alone (DEA +C) or lake water alone resulted in N_2_O production below the detection limit.

Denitrification enzyme activity (+C+N) was negatively correlated to redox, and positively correlated to normalized *nirS* copy numbers (Spearman’s ρ = -0.4762 and 0.4881, respectively; see next section for the results of *nirS* quantification) but significant correlations were not found with expected parameters, e.g., water column NO_2_^-^+NO_3_^-^ and TN (Supplementary Table S2). No measured lake parameters identified were significantly correlated to DEA+N (Supplementary Table S2).

### Quantitative PCR

**FIGURE 5:**
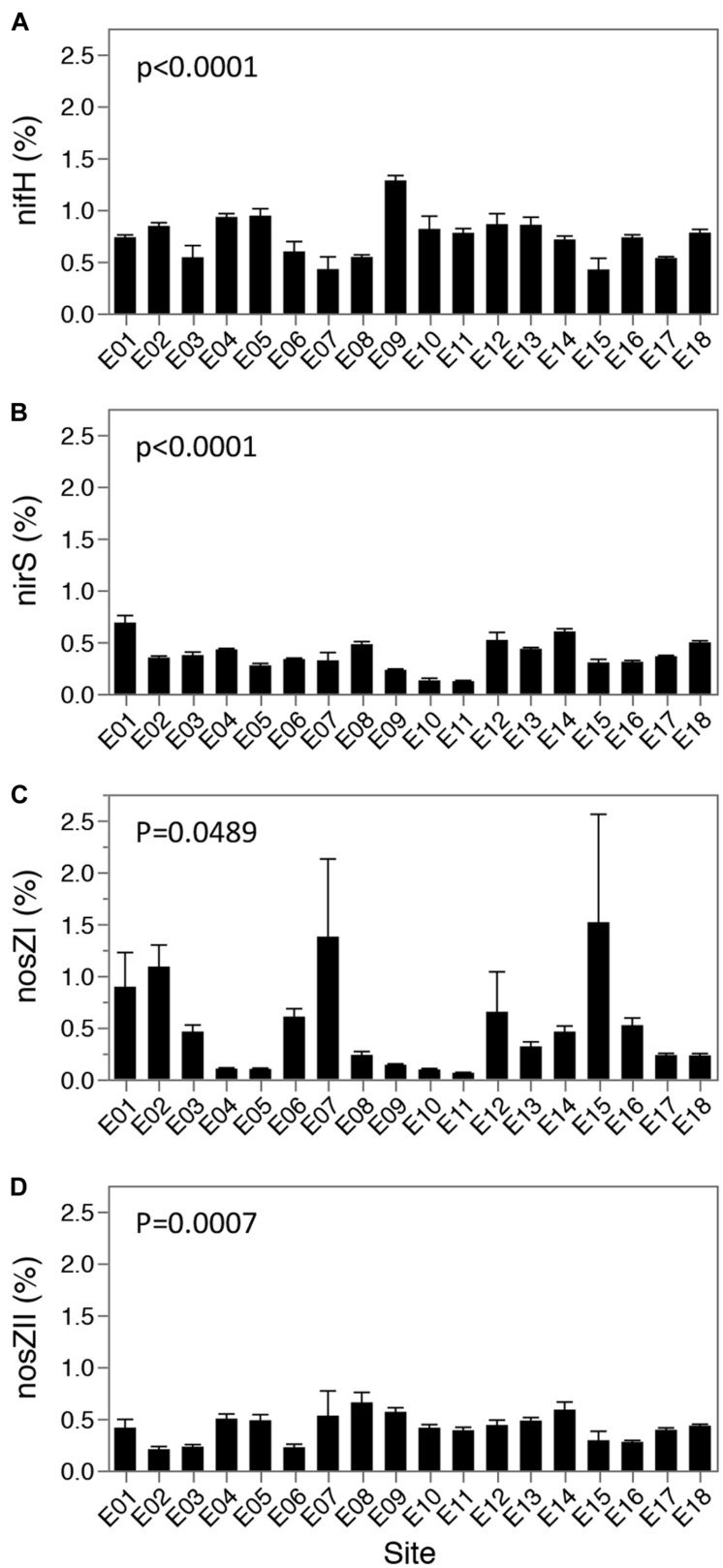
**Relative abundance (%) of nitrogen fixation and denitrifying genes to 16S rRNA gene abundances.** Values are shown as relative abundance (functional gene copies/16S rRNA gene copies), in percentage for N fixation (**(A)** *nifH* gene) and denitrification (**(B)** *nirS*, **(C)** *nosZI* and **(D)** *nosZII* gene). Values per site represent four biological replicates, as well as 4 technical replicates, and error bars indicate one std error of the mean. *P* values represent significance as assessed by one way ANOVA.

To determine spatial patterns, and changes correlated to physicochemical gradients, for functional groups involved in nitrogen cycling, functional genes involved in denitrification (i.e., loss of nitrogen) and nitrogen fixation (i.e., biologically linked inflow of nitrogen) were assessed via quantitative PCR (qPCR) of the *nirS*, *nosZI*, *nosZII*, and *nifH* genes. Functional gene copy numbers were normalized to the 16S rRNA gene abundance to account for any biases in DNA extraction, and to understand the proportion of the overall microbial community with the potential for nitrogen cycling functions. Normalized *nifH*, *nirS*, *nosZI*, and *nosZII* gene copy numbers changed significantly over the lake bed (One-way ANOVA: *p* < 0.0001, *nifH* and *nirS*; *p* = 0.0489, *nosZI*; *p* = 0.0007, *nosZII*), and at the majority of sites contributed to less than 1% of the total microbial community (**Figure 5**). No conserved pattern of nitrogen cycling gene distribution across the lake was identified (Supplementary Table S2; Supplementary Figure S7).

To understand the potential drivers and consequences of nitrogen cycling gene distribution throughout the lake, correlations were performed between lake parameters and normalized nitrogen cycling gene copy numbers. Spearman’s analysis identified correlations (Supplementary Table S2) with normalized *nifH* (above a 0.6 cutoff) to the interrelated variables: depth, silt %, sand % (Spearman’s ρ = 0.6636, 0.6677, -0.6326) and to NH_3_&NH_4_^+^ and TP (Spearman’s ρ = 0.6450, 0.6326). Similarly, normalized *nosZI* showed strong correlations to sand % and related variables including depth, silt %, clay % and bottom salinity (Spearman’s ρ = 0.7110, -0.7337, -0.6553, -0.6553, and -0.6718, respectively). Normalized *nirS* and *nosZII* values were unrelated to measured physical and chemical variables across the lake above the 0.6 Spearman’s cutoff (Supplementary Table S2).

### Microbial Community Composition

To determine spatial patterns, and changes correlated to physicochemical gradients, for the prokaryotic microbial community 16S rRNA gene amplicon sequencing was carried out. There was a 2.6-fold difference in observed OTUs across the lake (Supplementary Figure S8, One way ANOVA, *p* < 0.0001) with site richness ranging from 2362 observed OTUs at site E2 to 6239 at site E5. Richness was correlated to changes in sediment characteristics (sand%, clay%, silt%; Spearman’s ρ = -0.7424, 0.7610, 0.6959), with increasing richness associated to a shift from sandy to silty sediments. Other variables linked to sediment structure (depth, bottom salinity, organic matter %, *nifH*/16S, *nosZI*/16S), correlated to changes in richness (Supplementary Table S2). Richness was also strongly correlated to changes in normalized *nosZII* (Spearman’s ρ = 0.7424). Observed correlations with richness were conserved when substituted with Shannon diversity values (ANOVA *p* < 0.0001) but the strength of correlations to physical, chemical variables and biological variables were reduced (e.g., sand%, clay%, silt%; Spearman’s ρ = -0.6244, 0.5439, 0.6677).

**FIGURE 6:**
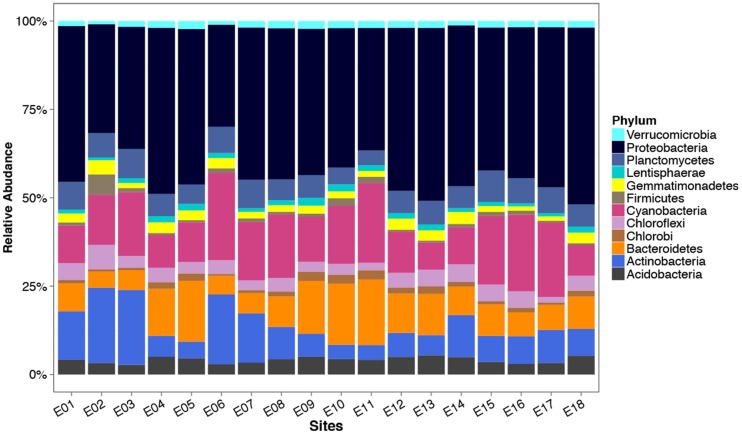
**Relative abundance of phyla within Lake Ellesmere as determined from 16S rRNA gene amplicon sequencing.** Bars represent the mean percentage of the microbial community attributable to different phyla within four site replicates for each of the 18 lake sites. Phyla representing less than 1% on average of the community at a site were excluded.

Abundance of identified phyla based on the 16S rRNA gene varied significantly between lake sites (One way Anova, *p* < 0.01; **Figure 6**) with the exception of a number of low abundance phyla (Archaeplastida, BHI80-139, Caldiserica, Candidate division TM7, WD272, SM2F11, SAR, LPD1-PA38, GA108, Fusobacteria, Dictyoglomi). Actinobacteria, Cyanobacteria, and Bacteroidetes were both abundant and variable across the lake with values increasing 5.6, 3.5, and 3.9-fold from the lowest abundance site to the highest. Proteobacteria and Cyanobacteria dominated the microbial community, making up on average 39 ± 5.4% and 14 ± 5.2% of the observed reads within a site (**Figure 6**). Actinobacteria, Bacteroidetes, Planctomycetes, Acidobacteria, and Chloroflexi also made up large proportions of the community contributing on average 9.5 ± 5.6%, 9.4 ± 4.0%, 6.3 ± 1.3%, 3.9 ± 0.85%, and 3.7 ± 1.2%, respectively. A fivefold enrichment of sequences assigned to the Firmicutes within a single site (E2) was observed, compared to the lake average (5.5% of all sequences in that site compared with 1.1% in the rest).

**FIGURE 7:**
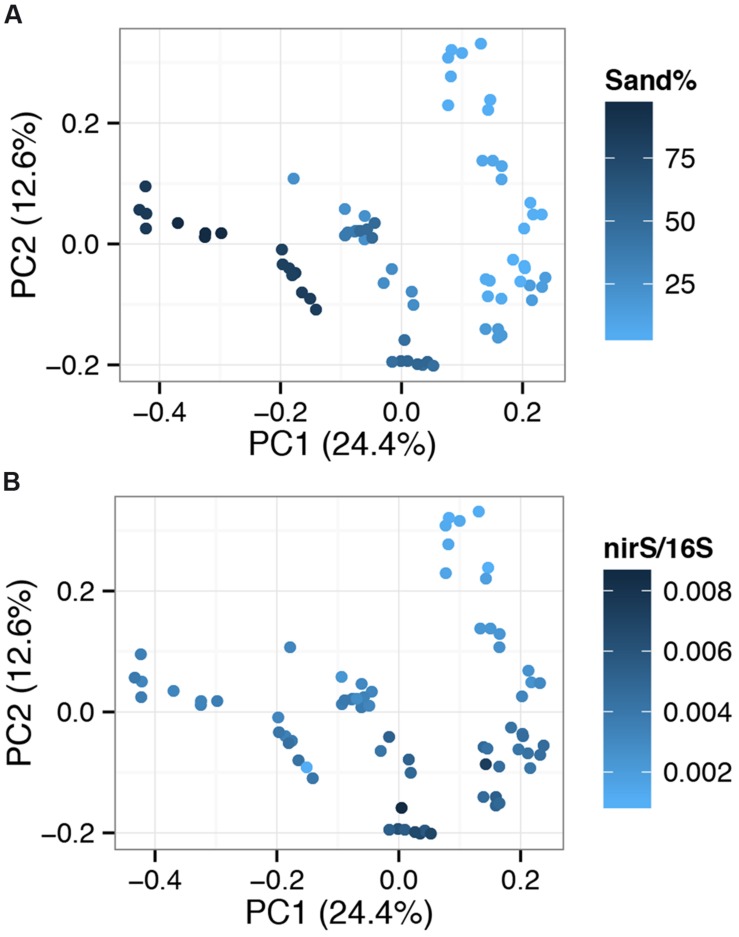
**Principal coordinate analysis (PCoA) plots based on OTU (Operational Taxonomic Units grouped at a 97% sequence similarity threshold) level relative abundance data, separated using Bray–Curtis dissimilarity.** Sites are color coded by sand% levels **(A)** or normalized *nirS* levels **(B)**. Percentages on axes show the variation explained by each component.

Principal coordinate analysis (PCoA) clustered sites into a gradient of community types with the first and second components explaining 24.4 and 12.6% of changes in community composition between sites (**Figure 7**). Physical parameters, primarily correlated to sediment structure (sand % and related variables; silt%, clay%, richness, diversity, organic matter%), were correlated to changes in community composition across component one (**Figure 7A**; Mantel test for sand% vs. community dissimilarity: *r* = 0.6318, *p* = 0.01). This was consistent independent of ordination method (Supplementary Figure S9). Component two was most strongly correlated to normalized *nirS* levels (**Figure 7B**). Spearman’s correlation identified a number of high abundance OTUs associated with increased normalized *nirS* levels (Supplementary Table S3).

## Discussion

### Impact of Nutrient and Freshwater Inflow

The impact of nutrient-rich freshwater inflow on lake chemistry, microbial community nitrogen cycling potential and whole microbial community structure was tested using areas of lowered salinity, and distance from sites of tributary inflow, as indicators of tributary influence. It was hypothesized that nutrient concentrations would form a gradient, along with salinity, from the two main freshwater inflows into the lake (the Selwyn and L2 rivers in the northern boundary), and that this gradient would create a selective force driving changes in microbial community structure at both the taxonomic and functional (nitrogen cycling gene abundance and denitrification enzymatic potential) level. Freshwater inflow, either from surface or ground water, can be identified by decreased water salinity around the north, west and eastern edges of the lake (Supplementary Figure S1) suggesting multiple significant inflows beyond the Selwyn and L2 rivers. These freshwater areas were associated with increases in NO_2_^-^+NO_3_^-^ levels at sites E1, 14 and 6 (Supplementary Figure S2; Supplementary Table S2). In contrast to the spatial distribution of the annual nitrate load (mean DIN load 646, 568, and 651 kg/day, respectively, for Selwyn River, L2 River and Harts Creek [unpublished report: Hamil and Schallenberg, (2013). Mechanisms that drive in-lake nutrient processing within Te Waihora/Lake Ellesmere: Inter-annual water quality. Report prepared for Whakaora Te Waihora, Christchurch, Canterbury, p. 54]), nitrate levels in the lake at the time of sampling were highest at sites E14 (near the mouth of Harts Creek) and E6 (at the Greenpark Sands) and not near two of the main predicted nitrate sources (the Selwyn River and L2). There is no obvious inflow located at the Greenpark Sands but the low relative salinity at this site suggests groundwater input at or near the site. Nitrate levels declined sharply with distance from these sites except for sites of moderate nitrate concentration at the eastern edge of the lake (sites E8 and E9). This suggests that either nitrate was rapidly consumed at these sites or that rapid dilution occurred, causing the nutrient gradient to be strongly localized. Alternatively, the observed spatial patterns of nutrient distributions in the lake could have been highly variable over the 3 days that sampling occurred suggesting that future studies would need to account for temporal changes. Other nutrients did not display strong spatial patterns associated with freshwater inflow, however, linear regressions show that TN, DRP, and possibly TP decreased with distance from the mouths of the Selwyn and L2 rivers (**Figure 2**). These patterns could have been influenced by multiple freshwater sources potentially carrying different nutrient loads [i.e., Selwyn is linked to DRP while Harts Creek and Greenpark Sands are associated to high NO_2_^-^+NO_3_^-^ levels (Supplementary Figure S1)].

Our analysis of lake chemistry provided no indication that the Selwyn or L2 rivers, our most important predicted sources of bulk nitrate were acting as nitrate sources at the time of sampling. Prior work monitoring long-term nutrient inflow (Hamil, unpublished) indicates highly episodic nitrate loading (generally in winter or spring) correlating with high water inflows. Sampling occurred during an extended period of low flows in the Selwyn River (average flow at the time of sampling was 1.81 m^3^s^-1^ compared to an annual average of 3.77 m^3^s^-1^ from 2009 to 2014), when a nutrient gradient from site E2 would be relatively poorly defined. Conversely, the L2 River and Harts creek were experiencing higher than average flows (3.31 and 1.74 m^3^s^-1^ compared to a yearly average of 2.30 and 1.55 m^3^s^-1^ from 2009 to 2014) but with no indication that the mouth of the L2 River (E3) was a source of nitrate. It is conceivable that our spatial mapping resolution was too low to detect nitrate inflow before it was consumed in the vicinity of the L2 inflow.

In the absence of strong nutrient loading, patterns of nutrient distribution within the lake are likely the result of intrinsic biogeochemical cycling and in lake mixing events driven by currents/wind events. Indeed, previous studies have demonstrated nutrient release from sediment induced by wind driven mixing (Schallenberg and Burns, 2004; Zhu et al., 2005; Wang et al., 2015). Regardless of the source of water column nutrients, no strong correlations with microbial community structure and functional potential were identified. Unfortunately, this study did not measure nutrient concentrations within sediment porewater thus we are unable to comment on the relationship between porewater and water column nutrient concentrations which might provide a more direct link to the benthic community. Such a finding may in fact indicate significant decoupling between the water column and sediment nutrient concentrations, limiting our ability to link sediment microbial community patterns to water column nutrients.

### Alternative Drivers of Microbial Community Structure at Phylogenetic and Functional Levels

**FIGURE 8:**
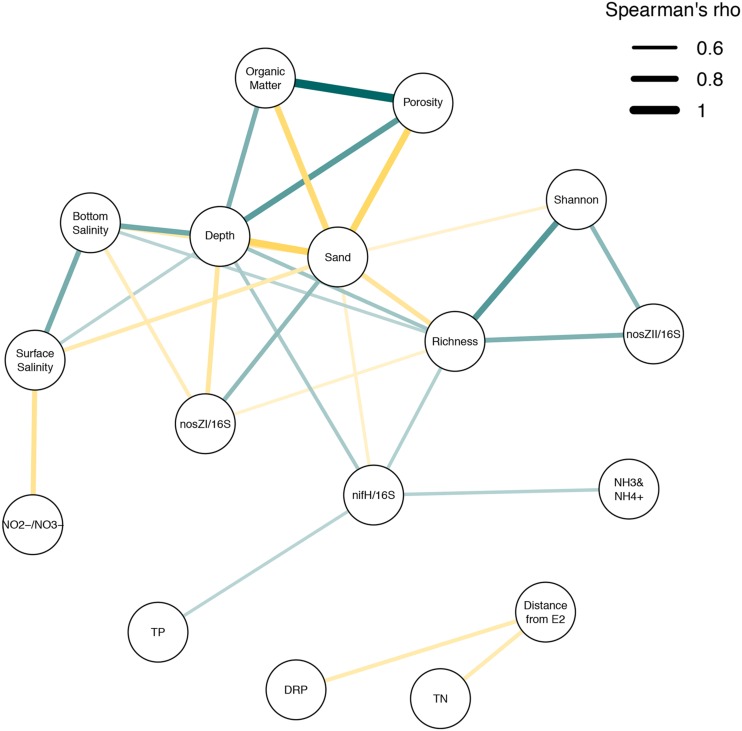
**Spearman’s correlation network showing major correlations between measured lake parameters.** Spearman’s analysis was carried out on lake metadata. Correlations above a 0.6 Spearmans rho (ρ) cut-off were included in analysis using qgraph and associated dependencies in R. Lines between nodes indicate an association between connected parameters. Node thickness and color intensity explains the strength of association (more intense color/thicker lines represent stronger ρ values. Line colors describe positive (blue) and negative (gold) correlations. TN, Total Nitrogen concentration; TP, Total Phosphorus concentration; NH_3_ and NH_4_^+^, sum of ammonia and ammonium; NO_2_^-^+NO_3_^-^, sum of nitrite and nitrate; DRP, Dissolved Reactive Phosphorus; Sand, percentage of sediment weight attributable to sand size particles (63–2000 μm); Organic Matter, percentage of sediment weight attributable to organic matter; Porosity, percentage of sediment weight attributable to porewater; Shannon, Shannon Diversity.

In the absence of strong externally derived nutrient influence, we examined the link between sediment physical and chemical variations across the lake bed and microbial functional and community composition. Finer sediments were found at deeper sites, probably reflecting the erosion of fine particles from shallower areas and resettling at deeper sites. Persistence of fine particles is likely further supported by protection from wind-induced resuspension in deeper sites. Most microbial community changes were strongly correlated to sediment characteristics, primarily physical factors (**Figures 7** and **8**) as has been observed in other studies (Dang et al., 2008, 2009; Giere, 2009; Lallias et al., 2014). These physical factors represent a more stable, and thus consistent selective pressure shaping microbial communities. This is in contrast to the more episodic nature of nutrient inflow from freshwater sources. In addition to increased temporal stability, these factors directly influence the habitat of the studied microbial communities which reside within sediments.

Denitrification enzyme activity analyses demonstrated that denitrification activity in lake sediments was primarily limited by the availability of nitrate and secondarily by labile carbon since denitrification was observed in the absence of added carbon but required addition of nitrogen. It indicates that an available pool of labile carbon is present, but limited. This suggests that a pulse of nitrate supplied to lake sediments, would increase anoxic conditions (Dang and Jiao, 2014) with a concomitant increase on denitrification activity. However, comparison of lake parameters to DEA results (phenotype) and qPCR on N cycling genes (genotype) confirmed the lack of influence of nutrient inflow on the microbial community. It is possible the spatial distribution of microorganisms is not controlled by the amount of either organic carbon or nitrogen alone, but by their ratio in the sediments (Dang et al., 2009; Yoon et al., 2015a,b). Correlations also suggest that DEA results are unlikely to be influenced by any measured physical factors. Alternatively, these factors could still be important but the potential (both DEA and DNA based measurements) of the system could have been uncoupled to the actual activity (*in situ* rates of denitrification and expression of genetic potential) at the time of sampling. Physical gradients had the strongest effect on normalized gene copy numbers for *nosZI* and *nifH*, which were correlated with sandy and silty sediments, respectively. Low p values observed for *nirS* and *nosZII* could suggest that other parameters are involved in the relative abundance of denitrifiers carrying these genes unlike observed in other studies (Dang et al., 2009) (e.g., pH). It is also possible that the lake was dominated by the alternative variant of the nitrite reductase [the Cu- containing NirK (Zumft, 1997) although *nirS* is expected to be dominant (Nogales et al., 2002; Dini-Andreote et al., 2016].

When functional gene data was related to total community composition, the distribution of *nosZII* in the lake was strongly associated with both richness and diversity (Supplementary Table S2). These in turn were linked to physical gradients, suggesting a niche preference for these organisms. However, *nosZII* abundance did not show a significant correlation with these physical gradients. It is possible that *nosZII* shares an unknown driver with richness and diversity that is unrelated to the observed physical factors. For example, Clade II nitrous oxide reducers have been shown to be taxonomically diverse, found within a larger group of organisms than their Clade I counterparts (Sanford et al., 2012; Jones et al., 2013). Prior work suggests *nosZ* abundance and denitrification are related to changes in grain sizes (Perryman et al., 2011; Deslippe et al., 2014). Although the importance of physical factors in *nosZ* gene distributions has been reported only in soils (Jones et al., 2014; Morales et al., 2015) it suggests particle size could be a conserved selective pressure across ecosystems, and indeed granulometry has been shown to infleunce microbial communities (Lallias et al., 2014).

Nutrient ratios (Larned and Schallenberg, 2006) as well as phytoplankton nutrient enhancement bioassays have shown that phytoplankton growth in the lake can be N-limited at times (Hawes and Ward, 1996; Schallenberg, unpublished data). Furthermore, nitrogen-fixing cyanobacteria, including *Nodularia spumigena* and *Anabaena* sp., sometimes bloom in the lake (Hughey et al., 2013). We measured nitrogenase gene abundances (*nifH*) in the lake sediment to determine the potential for nitrogen fixation to occur there. Nitrogen fixers on average only represented 0.74 percent of the microbial community within the lake sediments but a strong association between relative abundance of the gene and NH_3_ and NH_4_^+^ levels (0.645, *p* < 0.01) suggests that this may be sufficient to have an effect on water column nutrient levels. It cannot be discounted that measured *nifH* levels may include sedimented (i.e., buried) nitrogen fixing cyanobacteria from prior blooms in the lake. We are not aware of any published or unpublished estimates of N-fixation for the lake and we are, therefore, unable to say how important this N acquisition pathway may be for N cycling in the lake.

One additional consideration is the importance of functional groups when they represent a low proportion of the community. In our study all measured functional groups represented <2% of the community. Relative contributions (% of community) of functional groups tend to be low (Bowers et al., 2008; Dang et al., 2013; Sonthiphand et al., 2013; Harter et al., 2014; Saarenheimo et al., 2015; Zhou et al., 2016), unless ecosystems are particularly enriched for certain groups. These values are affected by the size and composition of the community. While it indicates a small contribution relative to the size of the community, absolute numbers, which reflect the actual size of the functional population, were high (Supplementary Table S4). Median copy numbers per gram of sediment for *nifH, nirS, nosZI*, and *nosZII* were 3.52E+08, 1.72E+08, 1.73E+08, and 2.38E+08, respectively. However, it is worth noting that these values represent the size of the population and does not constitute a direct measure of activity, but rather the potential in the community. While DNA measurements show correlation with processes, they are weak, indicating that RNA level measurement must be taken to account for actual rates (Rocca et al., 2015).

Although we were able to identify patterns for certain taxonomic groups, these reiterated factors linked to overall community composition which were primarily associated with physical factors (**Figure 8**), with sand% as the strongest driver. Further, we could not identify a clear pattern associated with clustering of sites based on the second PCoA component, but the strong correlation with *nirS* abundance suggest that some selective pressure is being applied that preferentially selects for certain functional groups. This could be other factors (e.g., pH) which were not measured in this study.

## Conclusion

Lake Ellesmere is influenced by freshwater inflows and these freshwater sources can influence the nutrient status of the lake. However, the measured levels of nitrate at the time of sampling were low for this lake and did not support the *a priori* hypothesis that nitrate inputs from the Selwyn and L2 rivers were strong drivers of nitrogen levels in the lake at the time of sampling. Thus our results represent the lower end of the influence that can be exerted by these sources. In the absence of a strong pulse of NO_2_^-^+NO_3_^-^, physical gradients dominated correlations associated to microbial community response both at the functional (measured as nitrogen cycling enzymatic [DEA] and genetic [DNA] potential) and phylogenetic level (measured at the total community level based on 16S rRNA gene profiles). However, higher nutrient levels (DRP and TN) were associated with sources of freshwater, suggesting agricultural influences were still detectable, but not driving community structuring. Regardless of drivers, detection of denitrification functional genes and DEA demonstrated that sediments would carry out denitrification during nitrate enrichment, potentially reducing availability of nitrogen and downstream eutrophic effects such as phytoplankton blooms. Additionally, a nitrogen fixing population could further be contributing to N levels in the lake, with ammonium levels strongly correlated to the abundance of this functional group. Despite our findings, our interpretation is limited due to the lack of temporal data and sampling at times of high nutrient inflow. Further, our measure of DEA only allows for a measure of the potential for the community to carry out denitrification under ideal conditions, but not the *in situ* rates of the process within the lake, so it can only be used to determine changes in potential and not actual denitrification. Despite this, a better understanding of the potential for denitrification and the interrelated factors affecting microbial communities in these complex ecosystems was achieved (**Figure 8**). Future work looking at the *in situ* rates of denitrification, the effect of wind driven mixing and the ultimate fate of incoming N would further add to our knowledge on ecosystem services and their regulators in these lagoon ecosystems.

## Author Contributions

SR and MH have contributed equally to this work. SR, JC, MS, and SEM contributed to the study design and contributed data to the paper. MH, SR, JC, and MS carried out sampling. MH and JC carried out the lab work and data analysis. MH, SR, and SEM wrote the paper. All authors edited the paper.

## Conflict of Interest Statement

The authors declare that the research was conducted in the absence of any commercial or financial relationships that could be construed as a potential conflict of interest.
