# Supplementary material for: Physical Factors Correlate to Microbial Community Structure and Nitrogen Cycling Gene Abundance in a Nitrate Fed Eutrophic Lagoon
Source: Front Microbiol. 2016 Oct 25;7:1691. doi: 10.3389/fmicb.2016.01691 (PMC5078687; doi:10.3389/fmicb.2016.01691)
Supplement: Supplementary file 1 [file Data_Sheet_1.DOCX]

Supplementary Material

Physical factors drive microbial community structure and denitrification in a nitrate fed eutrophic lagoon

Matthew Highton^1^†, Stéphanie Roosa^1^†, Josie Crawshaw^2^, Marc Schallenberg^3^ Sergio E. Morales^1^*

^1^Department of Microbiology and Immunology, Otago School of Medical Sciences, University of Otago, Dunedin, New Zealand.

^2^ Department of Marine Science, University of Otago, Dunedin, New Zealand.

^3^Department of Zoology, University of Otago, Dunedin, New Zealand

***** **Correspondence:** SE Morales, Department of Microbiology & Immunology, University of Otago, PO Box 56, Dunedin 9054, New Zealand

Tel.: +64 3 479 3140; fax: +64 3 479 8540

E-mail address: sergio.morales@otago.ac.nz

†These authors have contributed equally to this work.

# Supplemental Text

**Supplemental text 1: Full description of lake physical and chemical profiling**

Physical and chemical gradients within Lake Ellesmere’s sediment and water column were analyzed across 18 sites (Figure 1-3, S1-6). Lake depth was highly correlated (p<0.001) with physical and sediment chemistry parameters: sand%, silt%, clay%, porosity%, organic matter% (ρ= -0.88, 0.82, 0.89, 0.81, 0.74) (Table S2). Through the deeper central region and south eastern perimeter of the lake, sediments were predominantly composed of silt and clay with higher levels of porosity and organic matter, while the northern perimeter was dominated by sand (Figure S3). The bulk Eh of the top 4cm of sediment ranged from -20 to -400 mV, indicating reducing environments (Figure S4). Organic matter within the lake sediments was generally low (0.7 to 6.5%) with the exception of site E15 (21.7%), which contained a subsurface layer of peat (Figure S4).

Water column nutrient concentrations were mostly homogenous with large changes only observed within one or two sites (Figure S1), except for DRP and NH_3_&NH_4_^+^, which showed greater variability across the lake. The means and standard deviations of measured variables were: TN (2518 ± 397 µg/L), TP (230 ± 36 µg/L), NO_2_^-^+NO_3_^-^ (230 ± 36 µg/L), DRP (29 ± 11 µg/L), NH_3_&NH_4_^+^ (147 ± 21 µg/L), surface salinity (9.6 ± 1.8 ppt), bottom salinity (10.2 ± 0.7 ppt). TN and TP concentrations ranged from 2016 to 2846 µg/L and 198 to 256 µg/L respectively, excluding low anomalous readings at site E17 (1175 µg/L and 97.92 µg/L). Nitrate levels where relatively high at sites E14, E6 and E1 (320 µg/L, 277 µg/L and 104 µg/L respectively) but levels were lower than 60 µg/L at all other sites around the lake (Figure S1). Lake salinity (surface and bottom) formed a weak north-south gradient (Figure S1). A 6.5 ppt salinity deficit in the surface water compared to the bottom water was observed at site E14 and this vertical salinity differential was greater than at any other site.

PCA of physical and chemical data was used to identify correlations between measured lake variables and the major gradients across the lake. Factor loadings demonstrated that salinity and sediment physical characteristics tended to associate with the first principal component (PC1) explaining 35.1% of the variability between sites while other characteristics of the lake water were mainly associated with the second component (PC2), accounting for 19.4% of the variability (Figure S5A). Linear regressions between site scores and lake variables (Figure S5 B, C) further supported this conclusion. Significant correlations (p<0.0001) were found between PC1 and sand%, silt% and clay%, porosity %, and between PC2 and TN, TP, Secchi depth and DRP levels in the water column (TN, TP, p<0.0001; DRP, p=0.004; Secchi depth, p=0.0027). Segregation between components suggests that the water column nutrients TN, TP, DRP and water clarity (Secchi depth) at the time of sampling were not significantly related to the sediment characteristics (sand%, silt%, clay% and depth). Measures of sediment chemistry (Eh, organic matter%) and bottom water salinity showed potential relationships with the physical component.

Spearman’s rank correlation and linear regression was used to further explore relationships suggested by the PCA. It was found that sand% correlated with a host of other lake variables including silt%, clay%, depth%, porosity%, bottom salinity and organic matter% (Table S2). Water column nutrients showed a less consistent distribution and weaker correlations with other lake parameters (Table S2). Linear regression demonstrated a strong association between TN and TP (0.888, p<0.0001, Figure S6) but this was only weakly supported by Spearman’s correlations (ρ=0.49, p=0.04). Weak correlations were also identified between TN and DRP as well as between NH_3_&NH_4_^+^ and surface salinity (ρ=0.58, 0.52).

# Supplemental Figures and Tables

**Figure S1** Variability in water column chemical parameters (Surface Salinity, Bottom Salinity, TN, TP, DRP, NH_3_&NH_4_^+^, NO_2_^-^+NO_3_^-^) per site (left column) and spatially across Lake Ellesmere (centre column). Maps represent 18 data points interpolated by kriging.


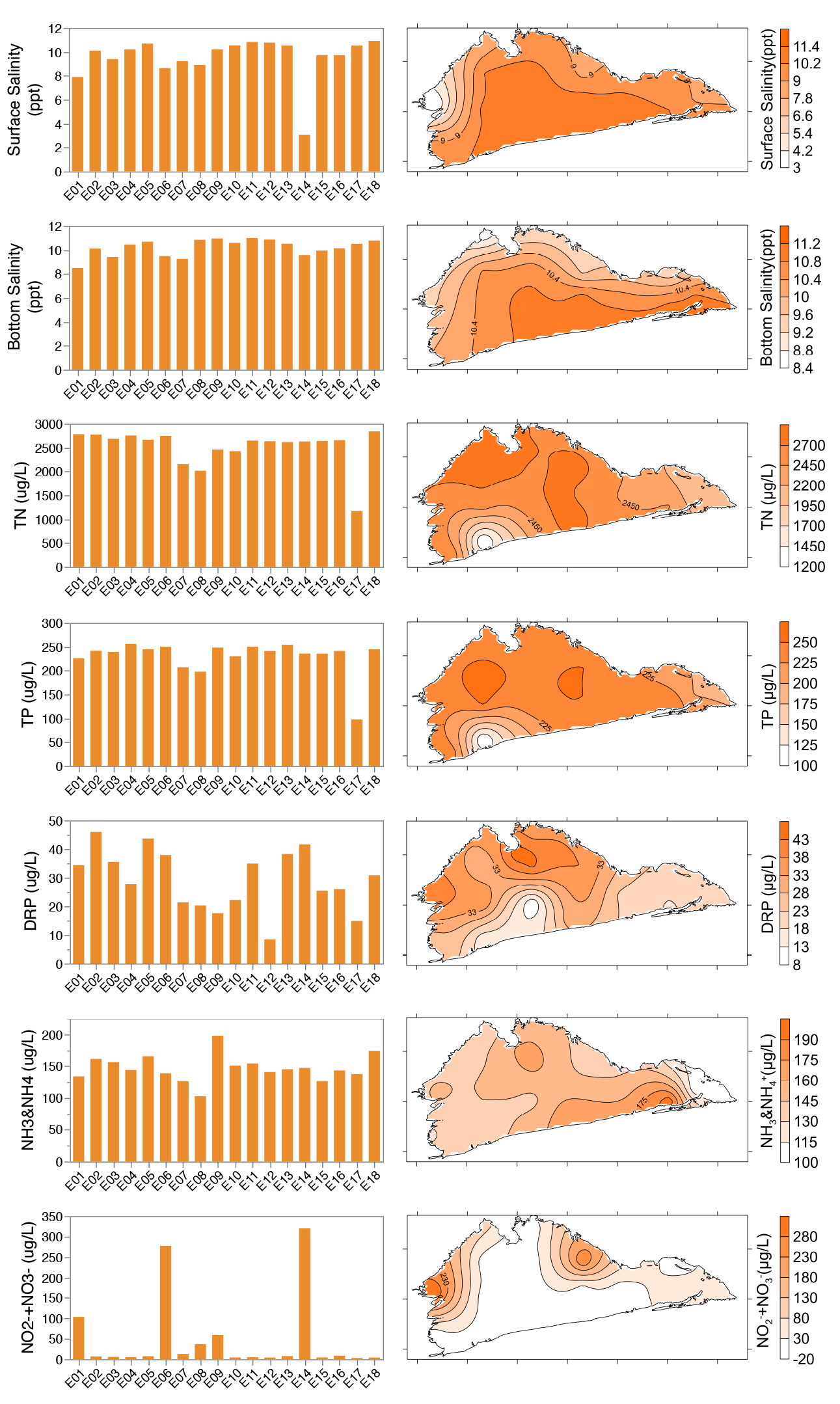


**Figure S2** Linear regression between surface salinity and NO_2_^-^+NO_3_^-^. Points represent a comparison between one-off measurements for variables at each of 18 lake sites. Shaded bars indicate confidence of fit.


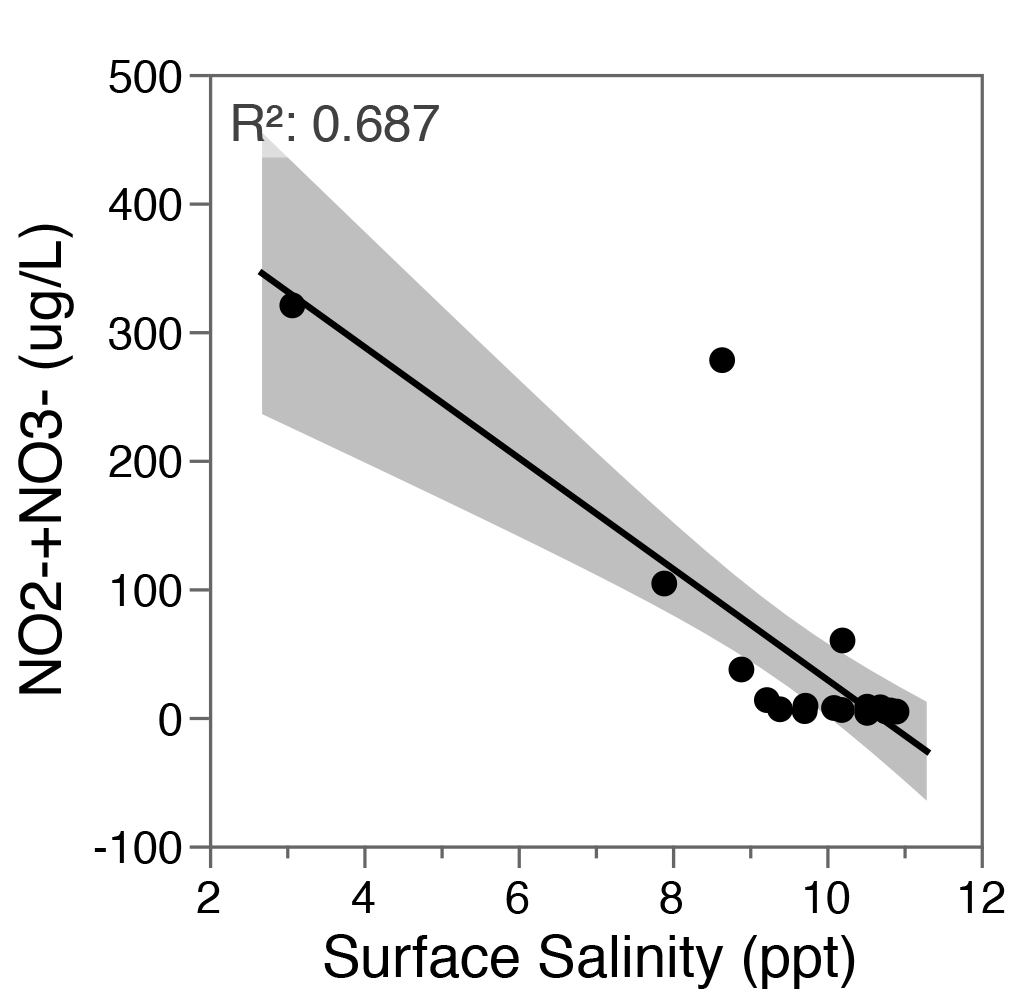


**Figure S3** Variability in physical parameters (depth, sand%, silt%, clay%, porosity%, Secchi, surface temperature, bottom temperature) per site (left column) and spatially across Lake Ellesmere (right column). Maps represent 18 data points interpolated by kriging.


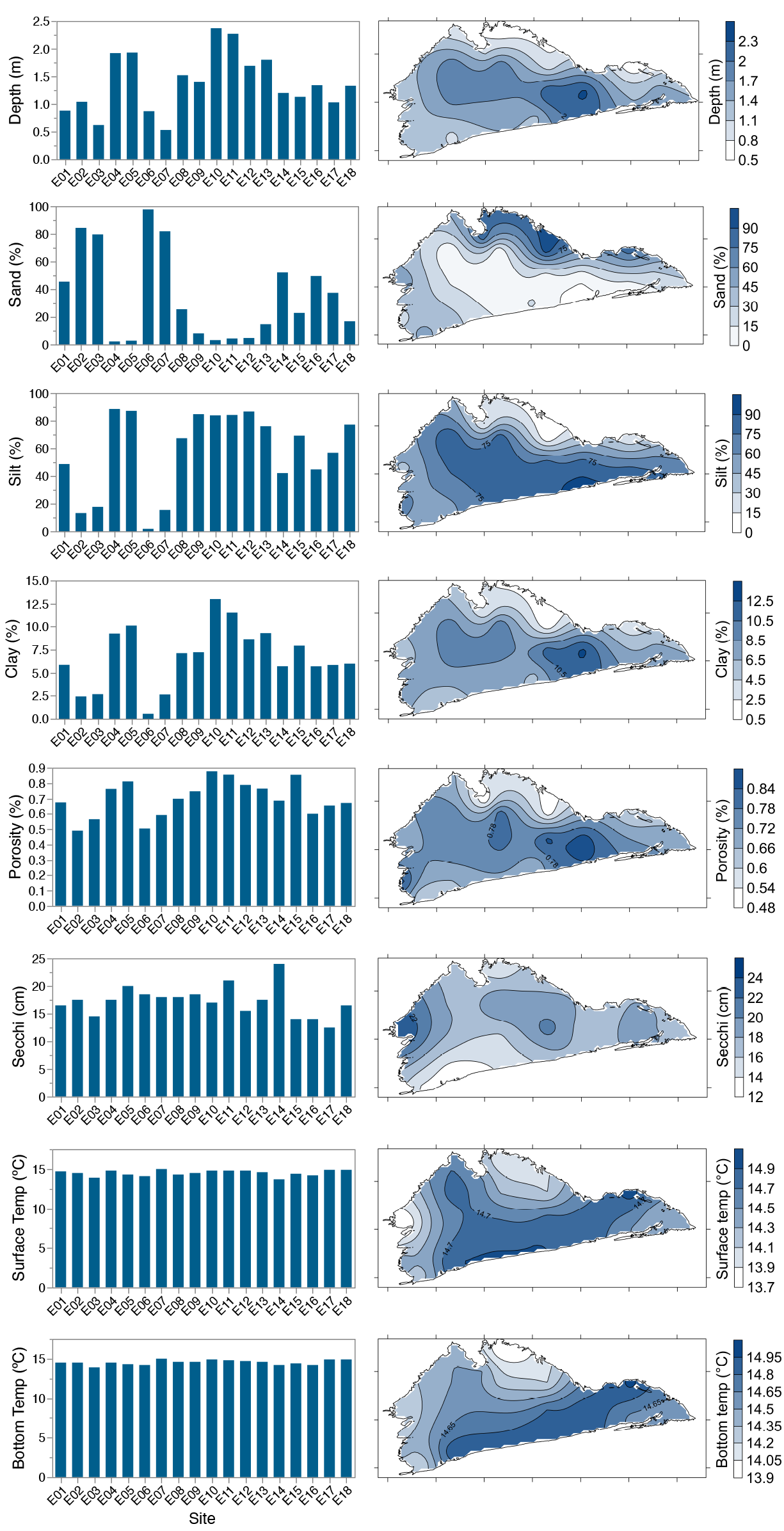


**Figure S4** Variability in sediment chemical parameters (redox [**A, B**], organic matter% [**C, D**]) per site (left column) and spatially across Lake Ellesmere (right column). Maps represent 18 data points interpolated by kriging.


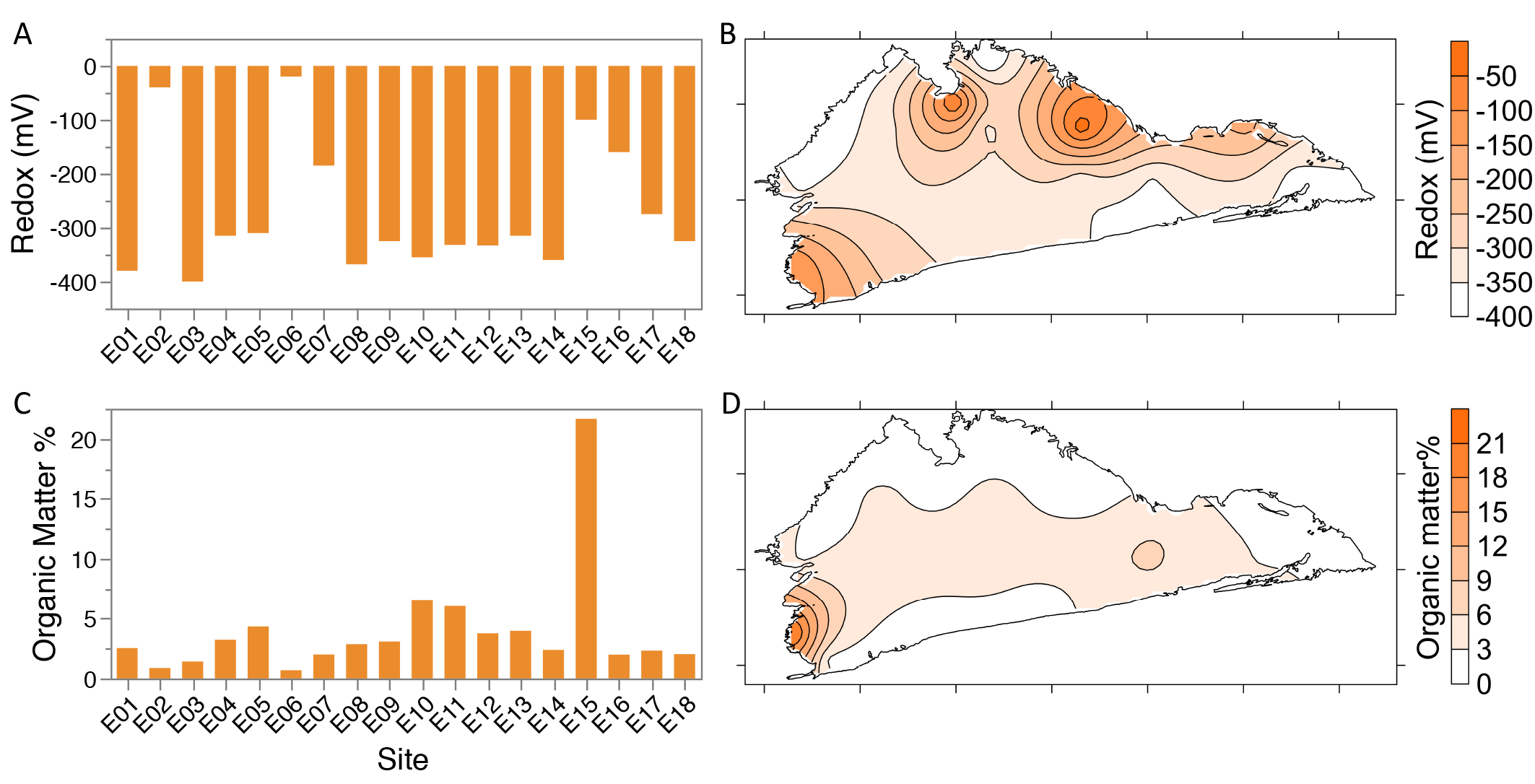


**Figure S5** Principal component analysis (PCA) of physical [blue] and chemical [orange] parameters within Lake Ellesmere. (**A**) Factor loadings based on separation of 18 lake sites by water chemistry (TN, TP, NH_3_&NH_4_^+^, NO_2_^-^+NO_3_^-^, DRP, surface salinity, bottom salinity), sediment chemistry (organic matter%, redox potential) and physical factors (depth, sand%, silt%, clay%, Secchi, porosity, bottom temperature, distance from site E2, distance from site E16). Axis percentage values indicate % of variability explained by that axis. (**B**) Linear regression of physical variables (Sand%, Clay%, Silt%) and PC1. (**C**) Linear regression of water chemistry variables (TN, TP, DRP) and PC2.


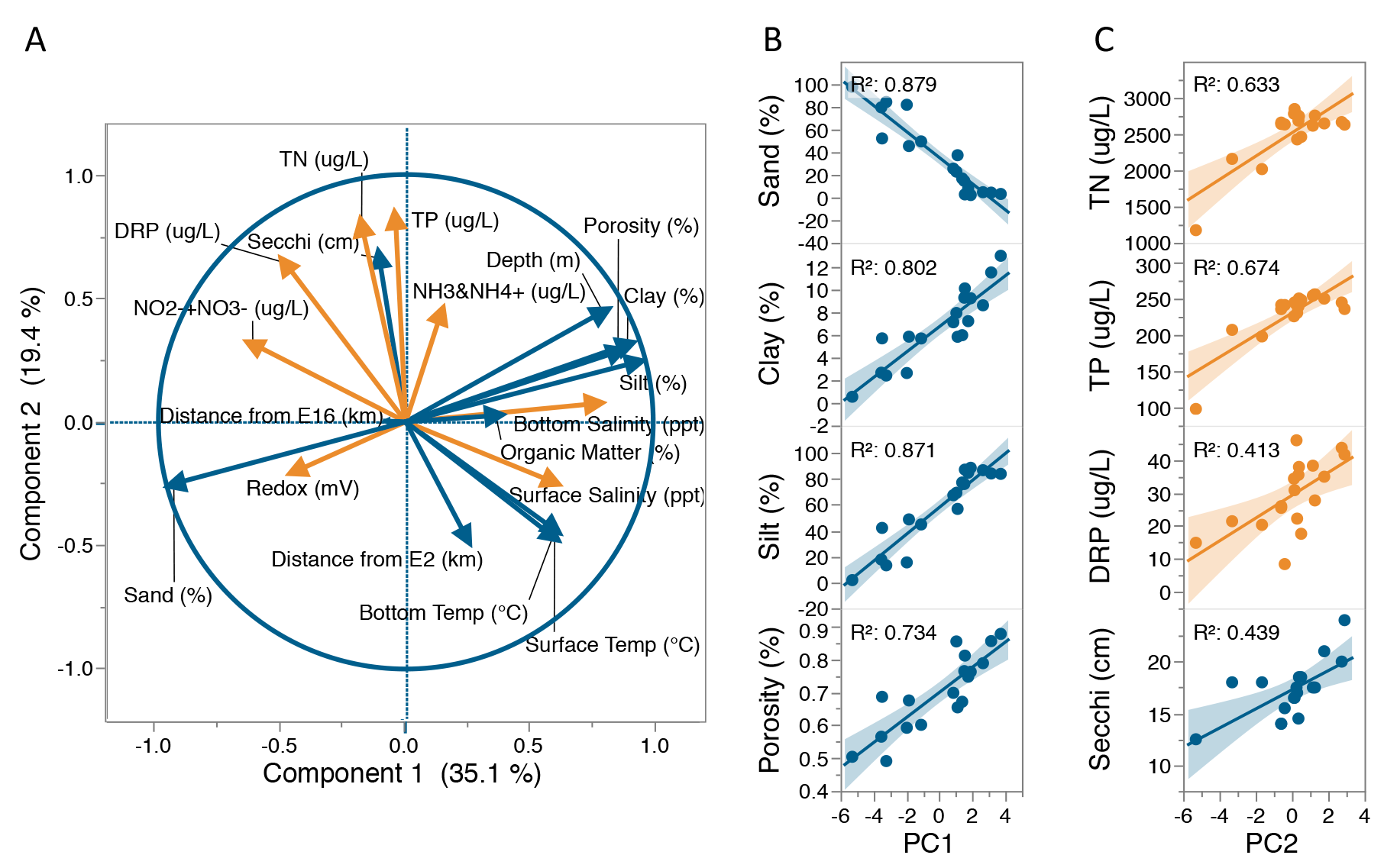


**Figure S6** Linear regression between TN and TP. Points represent a comparison between one-off measurements for variables at each of 18 lake sites. Shaded bars indicate confidence of fit.

**
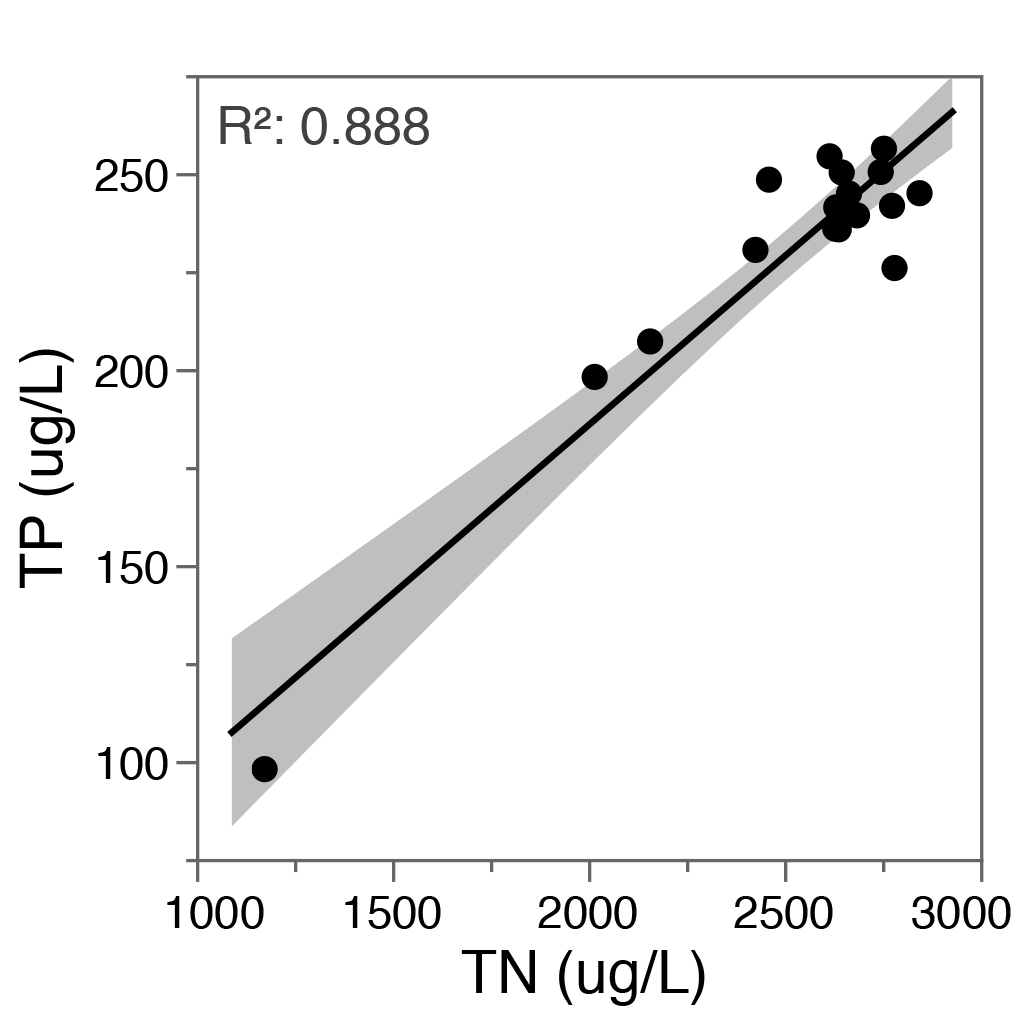
**

**Figure S7** Distribution of *nifH*, *nirS*, *nosZI* and *nosZII* nitrogen cycling genes throughout Lake Ellesmere sediments as a percentage of 16S copy number. Maps represent 18 data points (site averages) interpolated by kriging.


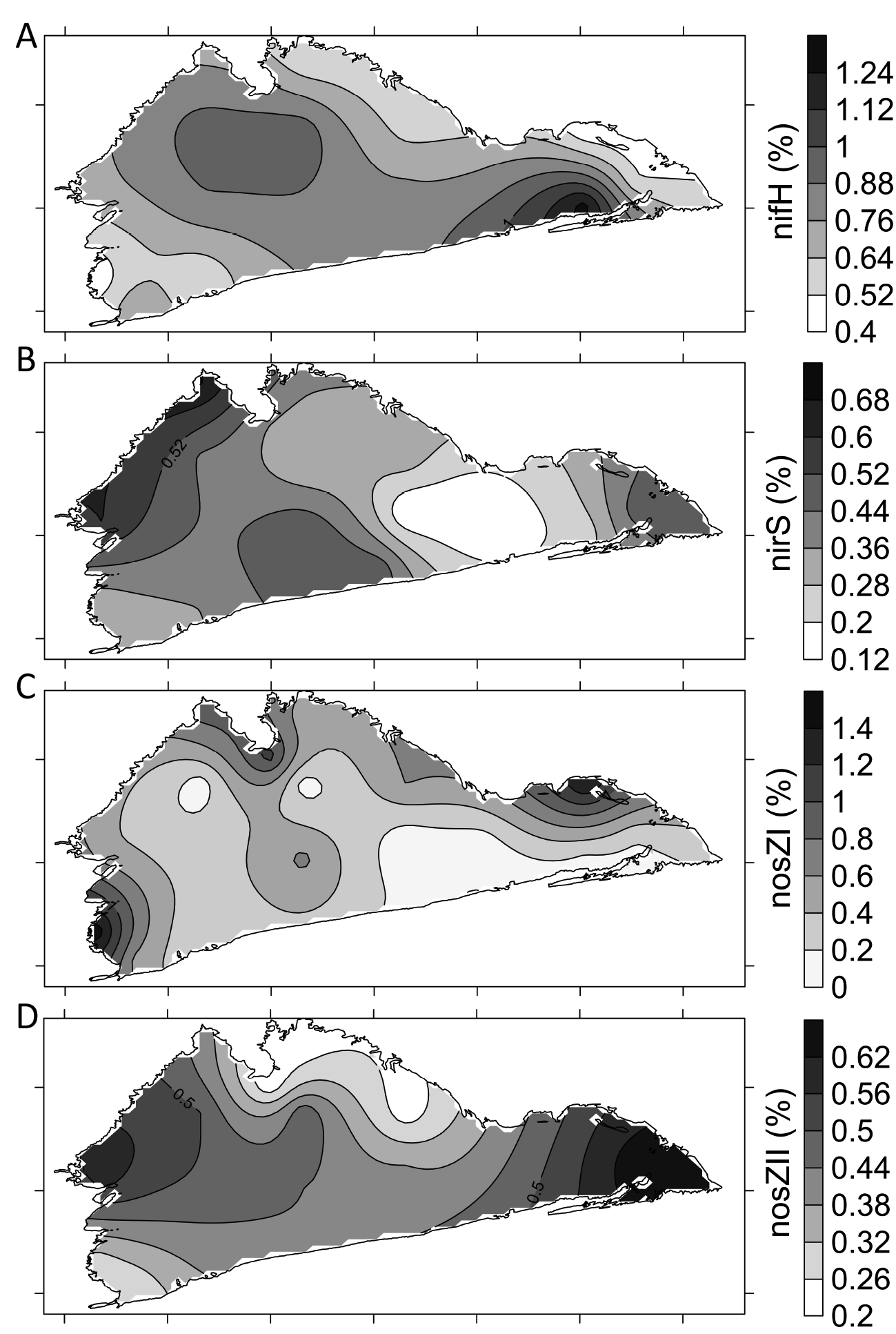


**Figure S8** Average sediment microbial richness (**A, B**) and shannon diversity (**C, D**) per site (left column) and across Lake Ellesmere. Error bars indicate one std error from the mean. p<0.0001, one-way ANOVA. Maps represent 18 data points (site averages) interpolated by kriging.


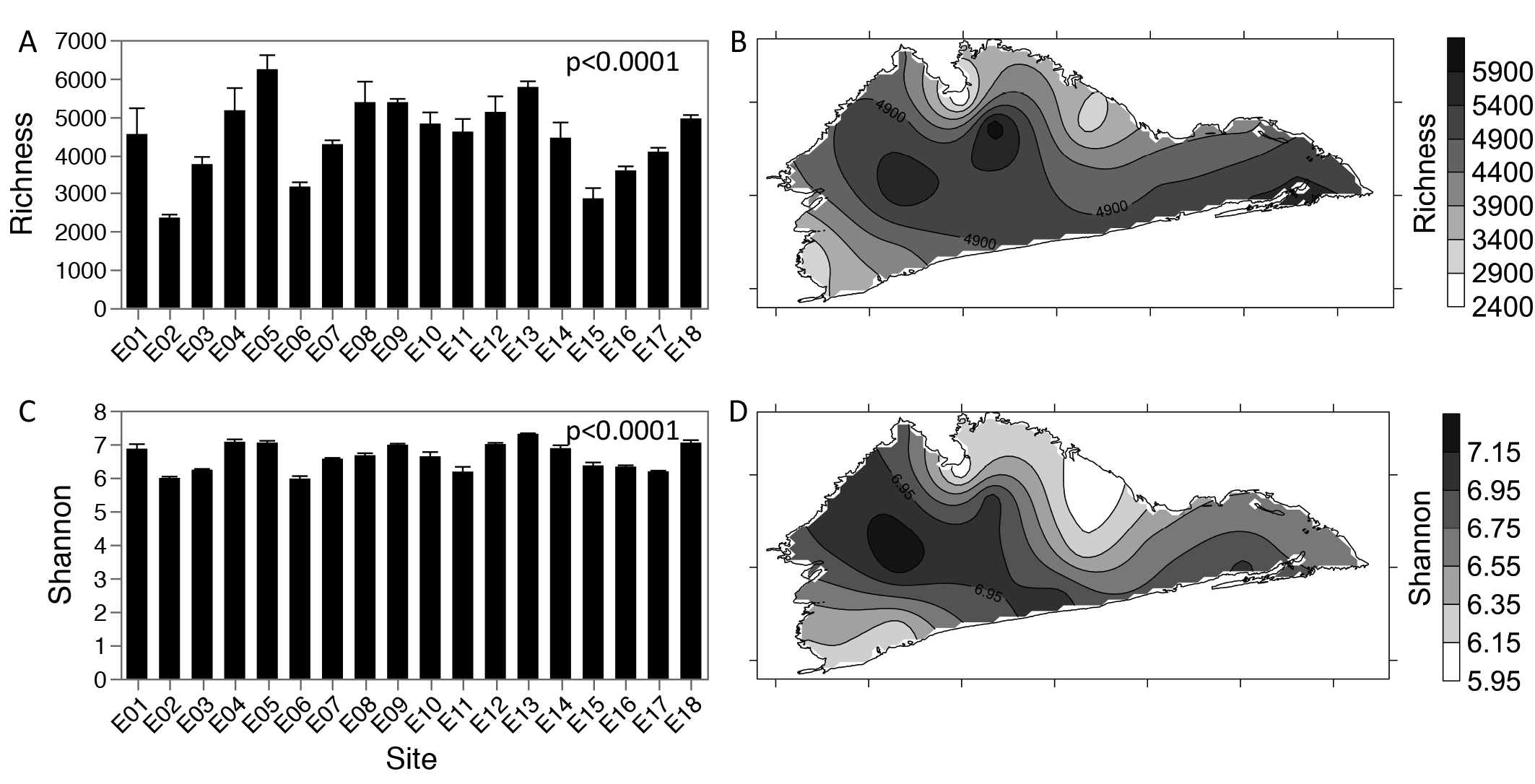


**Figure S9** Ordination of sites based on OTU level relative abundance data using canonical correspondence analysis (CCA), detrended correspondence analysis (DCA), multidimensional scaling (MDS), non-metric multidimensional scaling (NMDS), principal co-ordinate analysis (PCoA) and redundancy analysis (RDA). Sites are color coded by sand% levels (**A**) or normalized *nirS* gene abundance levels (**B**).


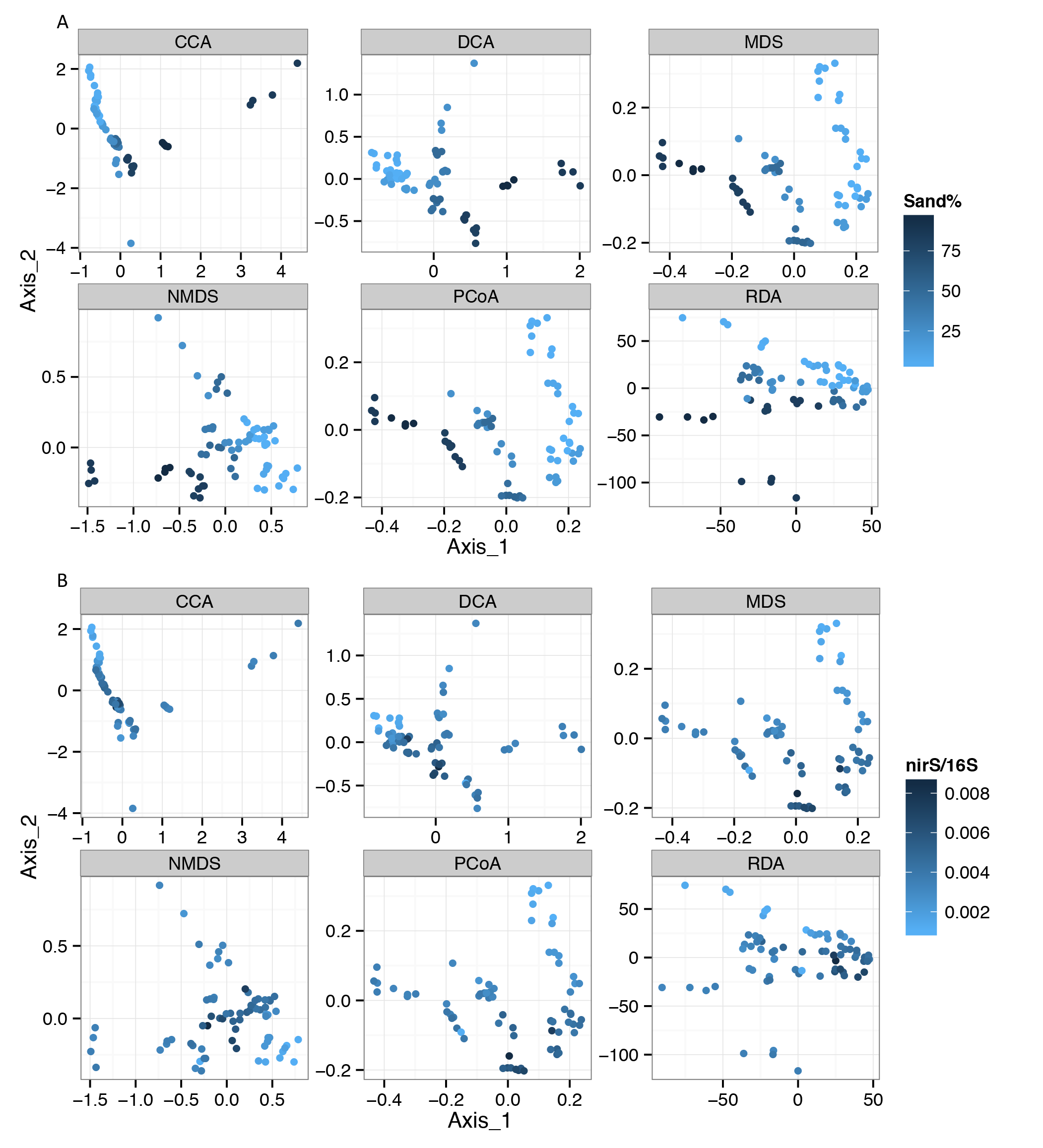


**Table S1** Lake sampling site co-ordinates (WGS84)

| WGS84 | Latitude | Longitude |
| --- | --- | --- |
| E1 | -43.72589509 | 172.4164276 |
| E2 | -43.748827 | 172.449359 |
| E3 | -43.728116 | 172.466425 |
| E4 | -43.761805 | 172.4159594 |
| E5 | -43.761955 | 172.4657566 |
| E6 | -43.762262 | 172.5155528 |
| E7 | -43.762412 | 172.6023561 |
| E8 | -43.787333 | 172.6270406 |
| E9 | -43.798591 | 172.60212 |
| E10 | -43.789411 | 172.5524849 |
| E11 | -43.789261 | 172.5152142 |
| E12 | -43.798044 | 172.4654401 |
| E13 | -43.797625 | 172.4157411 |
| E14 | -43.79405 | 172.3689313 |
| E15 | -43.833455 | 172.3656629 |
| E16 | -43.847326 | 172.3844225 |
| E17 | -43.833715 | 172.4153953 |

**Table S2** Spearman’s rank correlation of lake parameters

| Variable | by Variable | Spearman ρ | Prob>\|ρ\| |
| --- | --- | --- | --- |
| Porosity (%) | Organic Matter (%) | 0.9835 | <.0001 |
| 16S copy number | nosZII copy number | 0.967 | <.0001 |
| 16S copy number | nifH copy number | 0.9567 | <.0001 |
| Porosity (%) | Clay (%) | 0.9546 | <.0001 |
| Clay (%) | Organic Matter (%) | 0.934 | <.0001 |
| nosZII copy number | nifH copy number | 0.9278 | <.0001 |
| Bottom Temp (ºC) | Surface Temp (ºC) | 0.9041 | <.0001 |
| Clay (%) | Depth (m) | 0.8885 | <.0001 |
| Silt (%) | Clay (%) | 0.8865 | <.0001 |
| nifH copy number | Silt (%) | 0.8328 | <.0001 |
| Shannon | Richness | 0.8219 | <.0001 |
| Silt (%) | Depth (m) | 0.8184 | <.0001 |
| Porosity (%) | Depth (m) | 0.8142 | <.0001 |
| 16S copy number | Silt (%) | 0.8101 | <.0001 |
| Richness | nosZII copy number | 0.8085 | <.0001 |
| Porosity (%) | Silt (%) | 0.808 | <.0001 |
| nosZII copy number | Silt (%) | 0.7833 | 0.0001 |
| nifH copy number | Depth (m) | 0.7792 | 0.0001 |
| Richness | nifH copy number | 0.7785 | 0.0001 |
| Silt (%) | Organic Matter (%) | 0.775 | 0.0002 |
| Depth (m) | Bottom Salinity (ppt) | 0.7709 | 0.0002 |
| 16S copy number | Depth (m) | 0.7668 | 0.0002 |
| Richness | Silt (%) | 0.761 | 0.0002 |
| Bottom Salinity (ppt) | Surface Salinity (ppt) | 0.7599 | 0.0003 |
| Richness | 16S copy number | 0.7589 | 0.0003 |
| 16S copy number | Bottom Salinity (ppt) | 0.7523 | 0.0003 |
| 16S copy number | Clay (%) | 0.7523 | 0.0003 |
| Organic Matter (%) | Depth (m) | 0.7441 | 0.0004 |
| nosZII/16S | Richness | 0.7424 | 0.0004 |
| nifH copy number | Bottom Salinity (ppt) | 0.742 | 0.0004 |
| nosZII copy number | Depth (m) | 0.7399 | 0.0004 |
| Silt (%) | Bottom Salinity (ppt) | 0.7379 | 0.0005 |
| nifH copy number | Clay (%) | 0.7358 | 0.0005 |
| nosZII/16S | Shannon | 0.7193 | 0.0008 |
| nosZII copy number | Bottom Salinity (ppt) | 0.7172 | 0.0008 |
| nosZII copy number | Clay (%) | 0.711 | 0.0009 |
| nosZI/16S | Sand (%) | 0.711 | 0.0009 |
| nifH/16S | nifH copy number | 0.709 | 0.001 |
| Silt (%) | Surface Salinity (ppt) | 0.698 | 0.0013 |
| Richness | Clay (%) | 0.6959 | 0.0013 |
| Shannon | nirS copy number | 0.6883 | 0.0016 |
| nosZII copy number | nirS copy number | 0.6801 | 0.0019 |
| Richness | Depth (m) | 0.6794 | 0.0019 |
| Clay (%) | Bottom Salinity (ppt) | 0.6677 | 0.0025 |
| Shannon | Silt (%) | 0.6677 | 0.0025 |
| nifH/16S | Silt (%) | 0.6677 | 0.0025 |
| nifH/16S | Depth (m) | 0.6636 | 0.0027 |
| nifH copy number | Surface Salinity (ppt) | 0.6618 | 0.0028 |
| Richness | nirS copy number | 0.6608 | 0.0028 |
| 16S copy number | Surface Salinity (ppt) | 0.6536 | 0.0033 |
| nifH/16S | Richness | 0.6464 | 0.0038 |
| nifH/16S | NH3&NH4+ (ug/L) | 0.645 | 0.0038 |
| 16S copy number | nirS copy number | 0.6409 | 0.0042 |
| Clay (%) | Surface Salinity (ppt) | 0.6371 | 0.0045 |
| Depth (m) | Surface Salinity (ppt) | 0.636 | 0.0045 |
| Richness | Bottom Salinity (ppt) | 0.635 | 0.0046 |
| nifH/16S | TP (ug/L) | 0.6326 | 0.0048 |
| Porosity (%) | nifH copy number | 0.6202 | 0.006 |
| Porosity (%) | 16S copy number | 0.6202 | 0.006 |
| nirS copy number | nifH copy number | 0.6182 | 0.0063 |
| nosZII/16S | nirS copy number | 0.6078 | 0.0075 |
| Porosity (%) | nosZII copy number | 0.6037 | 0.008 |
| nosZII/16S | nosZII copy number | 0.5934 | 0.0094 |
| nifH/16S | Shannon | 0.5913 | 0.0097 |
| Porosity (%) | Bottom Salinity (ppt) | 0.5851 | 0.0107 |
| DRP (ug/L) | TN (ug/L) | 0.5769 | 0.0122 |
| nifH/16S | 16S copy number | 0.5728 | 0.013 |
| Porosity (%) | Richness | 0.57 | 0.0135 |
| Shannon | nifH copy number | 0.5686 | 0.0138 |
| NO2-+NO3- (ug/L) | Secchi (cm) | 0.5648 | 0.0146 |
| nirS copy number | Silt (%) | 0.5645 | 0.0147 |
| nifH/16S | nosZII copy number | 0.5604 | 0.0156 |
| 16S copy number | Organic Matter (%) | 0.5542 | 0.017 |
| nifH/16S | Bottom Salinity (ppt) | 0.5542 | 0.017 |
| NH3&NH4+ (ug/L) | TP (ug/L) | 0.5521 | 0.0175 |
| Surface Salinity (ppt) | Surface Temp (ºC) | 0.5463 | 0.019 |
| Shannon | Clay (%) | 0.5439 | 0.0196 |
| nifH/16S | Surface Salinity (ppt) | 0.541 | 0.0204 |
| nifH copy number | Organic Matter (%) | 0.5397 | 0.0208 |
| nosZII copy number | Organic Matter (%) | 0.5377 | 0.0214 |
| Richness | Organic Matter (%) | 0.5348 | 0.0222 |
| nosZI/16S | nosZI copy number | 0.5335 | 0.0226 |
| Bottom Temp (ºC) | Surface Salinity (ppt) | 0.5268 | 0.0247 |
| Shannon | nosZII copy number | 0.5232 | 0.0259 |
| NH3&NH4+ (ug/L) | Surface Salinity (ppt) | 0.5225 | 0.0261 |
| nifH/16S | Clay (%) | 0.517 | 0.028 |
| nosZII copy number | Surface Salinity (ppt) | 0.5152 | 0.0287 |
| Organic Matter (%) | Bottom Salinity (ppt) | 0.515 | 0.0287 |
| nifH copy number | NH3&NH4+ (ug/L) | 0.5067 | 0.0319 |
| Porosity (%) | Surface Salinity (ppt) | 0.4956 | 0.0365 |
| TP (ug/L) | TN (ug/L) | 0.4881 | 0.0399 |
| nirS/16S | DEA +C+N (ppmv h-1) | 0.4881 | 0.0399 |
| Shannon | Depth (m) | 0.4819 | 0.0428 |
| nosZI/16S | ng/µL_extracted | 0.4778 | 0.0449 |
| nosZI copy number | Sand (%) | 0.4757 | 0.046 |
| Shannon | 16S copy number | 0.4675 | 0.0504 |
| Porosity (%) | Shannon | 0.4613 | 0.054 |
| nosZII/16S | nifH copy number | 0.4592 | 0.0552 |
| Shannon | Organic Matter (%) | 0.4551 | 0.0577 |
| nosZII/16S | Secchi (cm) | 0.4539 | 0.0585 |
| Sand (%) | NO2-+NO3- (ug/L) | 0.453 | 0.059 |
| Organic Matter (%) | Surface Salinity (ppt) | 0.4502 | 0.0608 |
| nirS/16S | nirS copy number | 0.4489 | 0.0617 |
| DRP (ug/L) | TP (ug/L) | 0.4469 | 0.063 |
| nirS/16S | nosZI copy number | 0.4427 | 0.0658 |
| nosZII/16S | 16S copy number | 0.4427 | 0.0658 |
| nifH copy number | TP (ug/L) | 0.4407 | 0.0672 |
| Bottom Salinity (ppt) | Bottom Temp (ºC) | 0.4402 | 0.0675 |
| 16S copy number | NH3&NH4+ (ug/L) | 0.4324 | 0.0731 |
| DEA +C+N (ppmv h-1) | DEA +N (ppmv h-1) | 0.4324 | 0.0731 |
| Richness | Surface Salinity (ppt) | 0.4308 | 0.0743 |
| nosZII/16S | Silt (%) | 0.4241 | 0.0794 |
| Secchi (cm) | Distance from E16 (km) | 0.4207 | 0.0821 |
| nosZII copy number | Secchi (cm) | 0.4207 | 0.0821 |
| NH3&NH4+ (ug/L) | DRP (ug/L) | 0.418 | 0.0844 |
| DRP (ug/L) | Secchi (cm) | 0.4135 | 0.0881 |
| nosZII/16S | DEA +N (ppmv h-1) | 0.4097 | 0.0913 |
| DEA +C+N (ppmv h-1) | NO2-+NO3- (ug/L) | 0.4076 | 0.0931 |
| NH3&NH4+ (ug/L) | Bottom Salinity (ppt) | 0.3911 | 0.1085 |
| nosZII copy number | NH3&NH4+ (ug/L) | 0.3911 | 0.1085 |
| DEA +N (ppmv h-1) | Distance from E16 (km) | 0.3911 | 0.1085 |
| TP (ug/L) | Depth (m) | 0.3808 | 0.119 |
| 16S copy number | Bottom Temp (ºC) | 0.3736 | 0.1267 |
| nirS copy number | Clay (%) | 0.3725 | 0.1279 |
| nifH/16S | Porosity (%) | 0.3705 | 0.1302 |
| nosZII/16S | Distance from E2-E3 (m) | 0.3705 | 0.1302 |
| Richness | Secchi (cm) | 0.3681 | 0.1329 |
| Shannon | DEA +N (ppmv h-1) | 0.3643 | 0.1372 |
| nifH/16S | nirS copy number | 0.3643 | 0.1372 |
| nirS/16S | Shannon | 0.3643 | 0.1372 |
| nirS copy number | Depth (m) | 0.3622 | 0.1396 |
| DEA +C+N (ppmv h-1) | Distance from E16 (km) | 0.3622 | 0.1396 |
| TP (ug/L) | Secchi (cm) | 0.3617 | 0.1403 |
| TP (ug/L) | Surface Salinity (ppt) | 0.3562 | 0.1468 |
| NH3&NH4+ (ug/L) | TN (ug/L) | 0.354 | 0.1496 |
| nifH copy number | Secchi (cm) | 0.3523 | 0.1516 |
| Silt (%) | Surface Temp (ºC) | 0.3481 | 0.157 |
| nirS/16S | DEA +N (ppmv h-1) | 0.3457 | 0.16 |
| NH3&NH4+ (ug/L) | Depth (m) | 0.3416 | 0.1653 |
| Silt (%) | TP (ug/L) | 0.3375 | 0.1708 |
| nosZII copy number | Bottom Temp (ºC) | 0.3361 | 0.1727 |
| Clay (%) | Bottom Temp (ºC) | 0.334 | 0.1755 |
| nosZII/16S | Porosity (%) | 0.3333 | 0.1765 |
| nirS copy number | Bottom Salinity (ppt) | 0.3313 | 0.1793 |
| DRP (ug/L) | NO2-+NO3- (ug/L) | 0.3292 | 0.1822 |
| nosZI/16S | nirS/16S | 0.3271 | 0.1851 |
| nosZII/16S | Clay (%) | 0.3271 | 0.1851 |
| nosZII/16S | Organic Matter (%) | 0.3251 | 0.1881 |
| nifH copy number | Bottom Temp (ºC) | 0.3236 | 0.1902 |
| 16S copy number | Surface Temp (ºC) | 0.32 | 0.1955 |
| NO2-+NO3- (ug/L) | Distance from E16 (km) | 0.3189 | 0.1971 |
| Distance from E2-E3 (m) | Bottom Temp (ºC) | 0.3174 | 0.1994 |
| nifH/16S | Organic Matter (%) | 0.3168 | 0.2002 |
| nifH/16S | Secchi (cm) | 0.314 | 0.2045 |
| Silt (%) | NH3&NH4+ (ug/L) | 0.3127 | 0.2065 |
| Shannon | Bottom Salinity (ppt) | 0.3127 | 0.2065 |
| nosZI/16S | Redox | 0.311 | 0.2091 |
| Silt (%) | Bottom Temp (ºC) | 0.307 | 0.2153 |
| 16S copy number | Secchi (cm) | 0.3057 | 0.2173 |
| Richness | Bottom Temp (ºC) | 0.3051 | 0.2183 |
| nosZII/16S | Depth (m) | 0.3044 | 0.2193 |
| Distance from E2-E3 (m) | nosZI copy number | 0.3024 | 0.2226 |
| DEA +N (ppmv h-1) | nirS copy number | 0.3003 | 0.226 |
| nosZII/16S | DEA +C+N (ppmv h-1) | 0.3003 | 0.226 |
| nosZII/16S | Bottom Salinity (ppt) | 0.2982 | 0.2293 |
| Clay (%) | Surface Temp (ºC) | 0.2971 | 0.2311 |
| 16S copy number | TP (ug/L) | 0.2962 | 0.2327 |
| TP (ug/L) | Bottom Salinity (ppt) | 0.29 | 0.2431 |
| nosZII/16S | NO2-+NO3- (ug/L) | 0.29 | 0.2431 |
| nifH copy number | Surface Temp (ºC) | 0.2878 | 0.2468 |
| nosZII/16S | Bottom Temp (ºC) | 0.2862 | 0.2496 |
| Shannon | Surface Salinity (ppt) | 0.2819 | 0.2571 |
| nirS copy number | Surface Salinity (ppt) | 0.2767 | 0.2663 |
| Organic Matter (%) | Bottom Temp (ºC) | 0.2758 | 0.268 |
| NH3&NH4+ (ug/L) | Secchi (cm) | 0.2736 | 0.272 |
| nosZII/16S | nirS/16S | 0.2735 | 0.2722 |
| nirS copy number | Surface Temp (ºC) | 0.2681 | 0.2822 |
| Depth (m) | Secchi (cm) | 0.2674 | 0.2835 |
| Richness | DEA +N (ppmv h-1) | 0.2664 | 0.2853 |
| Richness | TP (ug/L) | 0.2654 | 0.2872 |
| Porosity (%) | Bottom Temp (ºC) | 0.2654 | 0.2872 |
| Shannon | TP (ug/L) | 0.2652 | 0.2875 |
| Bottom Salinity (ppt) | Surface Temp (ºC) | 0.2618 | 0.2939 |
| TP (ug/L) | Redox | 0.2614 | 0.2948 |
| DEA +N (ppmv h-1) | nosZI copy number | 0.2549 | 0.3073 |
| Porosity (%) | nirS copy number | 0.2549 | 0.3073 |
| nosZI/16S | NO2-+NO3- (ug/L) | 0.2528 | 0.3114 |
| nosZII/16S | nifH/16S | 0.2528 | 0.3114 |
| nifH/16S | TN (ug/L) | 0.2508 | 0.3155 |
| DEA +C+N (ppmv h-1) | nirS copy number | 0.2487 | 0.3196 |
| Distance from E2-E3 (m) | Bottom Salinity (ppt) | 0.2487 | 0.3196 |
| Richness | NH3&NH4+ (ug/L) | 0.2478 | 0.3215 |
| Sand (%) | Redox | 0.2469 | 0.3233 |
| DEA +N (ppmv h-1) | NO2-+NO3- (ug/L) | 0.2425 | 0.3322 |
| nosZII copy number | Surface Temp (ºC) | 0.241 | 0.3353 |
| Bottom Temp (ºC) | Distance from E16 (km) | 0.2404 | 0.3366 |
| DEA +C+N (ppmv h-1) | Sand (%) | 0.2384 | 0.3408 |
| nirS/16S | Sand (%) | 0.2384 | 0.3408 |
| DEA +C+N (ppmv h-1) | nosZI copy number | 0.2343 | 0.3495 |
| Sand (%) | DRP (ug/L) | 0.2281 | 0.3627 |
| Organic Matter (%) | Surface Temp (ºC) | 0.2265 | 0.3661 |
| Clay (%) | TP (ug/L) | 0.226 | 0.3672 |
| nosZI copy number | NO2-+NO3- (ug/L) | 0.226 | 0.3672 |
| Depth (m) | Bottom Temp (ºC) | 0.2217 | 0.3767 |
| nirS/16S | TN (ug/L) | 0.2198 | 0.3808 |
| nosZII/16S | Distance from E16 (km) | 0.2198 | 0.3808 |
| nirS copy number | Organic Matter (%) | 0.2178 | 0.3854 |
| Clay (%) | NH3&NH4+ (ug/L) | 0.2136 | 0.3947 |
| nosZII copy number | TP (ug/L) | 0.2136 | 0.3947 |
| Richness | Distance from E16 (km) | 0.2075 | 0.4086 |
| Richness | Surface Temp (ºC) | 0.2017 | 0.4223 |
| Secchi (cm) | Bottom Salinity (ppt) | 0.201 | 0.4238 |
| Porosity (%) | Surface Temp (ºC) | 0.1995 | 0.4274 |
| Shannon | NH3&NH4+ (ug/L) | 0.1971 | 0.4331 |
| nirS copy number | Bottom Temp (ºC) | 0.1946 | 0.4391 |
| DEA +N (ppmv h-1) | Secchi (cm) | 0.1896 | 0.451 |
| ng/µL_extracted | Sand (%) | 0.1889 | 0.4529 |
| Shannon | Surface Temp (ºC) | 0.1725 | 0.4937 |
| Distance from E2-E3 (m) | Organic Matter (%) | 0.1703 | 0.4993 |
| Distance from E2-E3 (m) | Porosity (%) | 0.1703 | 0.4993 |
| ng/µL_extracted | Organic Matter (%) | 0.1682 | 0.5046 |
| ng/µL_extracted | nosZI copy number | 0.1682 | 0.5046 |
| Richness | DEA +C+N (ppmv h-1) | 0.1662 | 0.5097 |
| nosZII copy number | Distance from E16 (km) | 0.1662 | 0.51 |
| nifH/16S | DRP (ug/L) | 0.16 | 0.5261 |
| nosZII/16S | nosZI copy number | 0.1579 | 0.5315 |
| Distance from E2-E3 (m) | nosZII copy number | 0.1517 | 0.5479 |
| nirS/16S | NO2-+NO3- (ug/L) | 0.1496 | 0.5534 |
| Porosity (%) | Secchi (cm) | 0.1492 | 0.5545 |
| Depth (m) | Surface Temp (ºC) | 0.1403 | 0.5788 |
| Porosity (%) | ng/µL_extracted | 0.1393 | 0.5814 |
| Shannon | Bottom Temp (ºC) | 0.1384 | 0.5839 |
| nosZII/16S | Surface Temp (ºC) | 0.1382 | 0.5845 |
| DEA +N (ppmv h-1) | TN (ug/L) | 0.1373 | 0.5871 |
| DEA +C+N (ppmv h-1) | Secchi (cm) | 0.1358 | 0.5912 |
| Shannon | Secchi (cm) | 0.1326 | 0.5998 |
| nosZI/16S | TN (ug/L) | 0.129 | 0.6099 |
| nosZI copy number | Redox | 0.125 | 0.6212 |
| Clay (%) | Secchi (cm) | 0.1244 | 0.623 |
| nosZI copy number | nirS copy number | 0.1228 | 0.6273 |
| DRP (ug/L) | Redox | 0.1219 | 0.6299 |
| Shannon | DEA +C+N (ppmv h-1) | 0.1146 | 0.6508 |
| Porosity (%) | TP (ug/L) | 0.1125 | 0.6568 |
| nifH/16S | Surface Temp (ºC) | 0.1112 | 0.6605 |
| nirS copy number | NH3&NH4+ (ug/L) | 0.1063 | 0.6746 |
| nirS/16S | Richness | 0.1012 | 0.6895 |
| nirS copy number | Secchi (cm) | 0.0984 | 0.6975 |
| Sand (%) | TN (ug/L) | 0.096 | 0.7048 |
| Distance from E2-E3 (m) | 16S copy number | 0.096 | 0.7048 |
| Organic Matter (%) | Secchi (cm) | 0.0943 | 0.7097 |
| Sand (%) | Distance from E16 (km) | 0.0918 | 0.717 |
| TN (ug/L) | Redox | 0.0878 | 0.729 |
| DEA +N (ppmv h-1) | Sand (%) | 0.0857 | 0.7354 |
| Porosity (%) | NH3&NH4+ (ug/L) | 0.0857 | 0.7354 |
| DEA +N (ppmv h-1) | DRP (ug/L) | 0.0815 | 0.7478 |
| Distance from E2-E3 (m) | Surface Temp (ºC) | 0.081 | 0.7492 |
| Silt (%) | Secchi (cm) | 0.0808 | 0.7499 |
| Distance from E2-E3 (m) | nifH copy number | 0.0795 | 0.754 |
| DEA +N (ppmv h-1) | nosZII copy number | 0.0733 | 0.7726 |
| Distance from E2-E3 (m) | Depth (m) | 0.0733 | 0.7726 |
| nirS copy number | TP (ug/L) | 0.0712 | 0.7789 |
| Shannon | nosZI copy number | 0.0712 | 0.7789 |
| nosZI/16S | DEA +N (ppmv h-1) | 0.0691 | 0.7851 |
| nifH/16S | Bottom Temp (ºC) | 0.0656 | 0.7961 |
| Distance from E2-E3 (m) | NO2-+NO3- (ug/L) | 0.065 | 0.7977 |
| Distance from E2-E3 (m) | Redox | 0.063 | 0.8038 |
| Surface Temp (ºC) | Distance from E16 (km) | 0.0623 | 0.8059 |
| Distance from E2-E3 (m) | Richness | 0.0609 | 0.8102 |
| Distance from E2-E3 (m) | Clay (%) | 0.0609 | 0.8103 |
| TP (ug/L) | Organic Matter (%) | 0.0568 | 0.823 |
| DEA +C+N (ppmv h-1) | TN (ug/L) | 0.0568 | 0.823 |
| DEA +C+N (ppmv h-1) | nosZII copy number | 0.0547 | 0.8293 |
| nirS/16S | DRP (ug/L) | 0.0547 | 0.8293 |
| Redox | Surface Temp (ºC) | 0.0525 | 0.836 |
| NO2-+NO3- (ug/L) | TP (ug/L) | 0.0506 | 0.8421 |
| 16S copy number | Distance from E16 (km) | 0.0485 | 0.8484 |
| nosZI/16S | DEA +C+N (ppmv h-1) | 0.0485 | 0.8484 |
| Redox | Surface Salinity (ppt) | 0.0429 | 0.8658 |
| nosZI copy number | Surface Temp (ºC) | 0.0384 | 0.8796 |
| Bottom Salinity (ppt) | Distance from E16 (km) | 0.0382 | 0.8804 |
| NH3&NH4+ (ug/L) | Distance from E16 (km) | 0.0382 | 0.8804 |
| nirS copy number | TN (ug/L) | 0.0382 | 0.8804 |
| Distance from E2-E3 (m) | Silt (%) | 0.0299 | 0.9062 |
| NH3&NH4+ (ug/L) | Organic Matter (%) | 0.0196 | 0.9384 |
| nifH/16S | Distance from E16 (km) | 0.0196 | 0.9384 |
| ng/µL_extracted | Clay (%) | 0.0175 | 0.9449 |
| NO2-+NO3- (ug/L) | TN (ug/L) | 0.0134 | 0.9579 |
| nirS/16S | ng/µL_extracted | 0.0114 | 0.9643 |
| TN (ug/L) | Surface Salinity (ppt) | 0.0103 | 0.9676 |
| nosZI copy number | Bottom Temp (ºC) | 0.0073 | 0.9771 |
| Distance from E2-E3 (m) | Shannon | 0.0072 | 0.9773 |
| Shannon | TN (ug/L) | 0.0052 | 0.9838 |
| Distance from E2-E3 (m) | ng/µL_extracted | 0.0052 | 0.9838 |
| nosZI/16S | DRP (ug/L) | 0.0031 | 0.9903 |
| Richness | NO2-+NO3- (ug/L) | 0 | 1 |
| nifH copy number | Distance from E16 (km) | -0.0031 | 0.9903 |
| Shannon | NO2-+NO3- (ug/L) | -0.0093 | 0.9708 |
| DEA +C+N (ppmv h-1) | nifH copy number | -0.0134 | 0.9579 |
| Distance from E2-E3 (m) | nirS copy number | -0.0134 | 0.9579 |
| DEA +C+N (ppmv h-1) | 16S copy number | -0.0237 | 0.9255 |
| Distance from E2-E3 (m) | Sand (%) | -0.0237 | 0.9255 |
| nosZI/16S | Distance from E2-E3 (m) | -0.0237 | 0.9255 |
| DEA +N (ppmv h-1) | NH3&NH4+ (ug/L) | -0.0258 | 0.9191 |
| DEA +C+N (ppmv h-1) | TP (ug/L) | -0.0258 | 0.9191 |
| ng/µL_extracted | DRP (ug/L) | -0.032 | 0.8997 |
| Distance from E2-E3 (m) | Distance from E16 (km) | -0.032 | 0.8997 |
| nirS/16S | Surface Temp (ºC) | -0.0343 | 0.8926 |
| DEA +C+N (ppmv h-1) | NH3&NH4+ (ug/L) | -0.0361 | 0.8869 |
| Distance from E2-E3 (m) | DEA +C+N (ppmv h-1) | -0.0382 | 0.8804 |
| DRP (ug/L) | Depth (m) | -0.0402 | 0.874 |
| ng/µL_extracted | Bottom Temp (ºC) | -0.0406 | 0.873 |
| ng/µL_extracted | Surface Temp (ºC) | -0.0426 | 0.8667 |
| DEA +N (ppmv h-1) | nifH copy number | -0.0444 | 0.8612 |
| nifH/16S | NO2-+NO3- (ug/L) | -0.0464 | 0.8548 |
| NO2-+NO3- (ug/L) | Redox | -0.0475 | 0.8515 |
| Shannon | DRP (ug/L) | -0.0485 | 0.8484 |
| nosZII/16S | Surface Salinity (ppt) | -0.0496 | 0.8452 |
| DEA +N (ppmv h-1) | Silt (%) | -0.0506 | 0.8421 |
| Secchi (cm) | Bottom Temp (ºC) | -0.0533 | 0.8337 |
| TN (ug/L) | Secchi (cm) | -0.0539 | 0.8318 |
| Redox | Bottom Temp (ºC) | -0.0552 | 0.8278 |
| Silt (%) | TN (ug/L) | -0.063 | 0.804 |
| ng/µL_extracted | DEA +N (ppmv h-1) | -0.065 | 0.7977 |
| Secchi (cm) | Redox | -0.0664 | 0.7935 |
| ng/µL_extracted | Distance from E16 (km) | -0.0671 | 0.7914 |
| nirS/16S | nifH/16S | -0.0671 | 0.7914 |
| DEA +N (ppmv h-1) | Bottom Temp (ºC) | -0.0676 | 0.7897 |
| ng/µL_extracted | TN (ug/L) | -0.0691 | 0.7851 |
| Clay (%) | Distance from E16 (km) | -0.0712 | 0.7789 |
| DEA +N (ppmv h-1) | 16S copy number | -0.0712 | 0.7789 |
| DEA +N (ppmv h-1) | Surface Temp (ºC) | -0.0727 | 0.7743 |
| nosZII/16S | NH3&NH4+ (ug/L) | -0.0815 | 0.7478 |
| nifH/16S | DEA +C+N (ppmv h-1) | -0.0857 | 0.7354 |
| Distance from E2-E3 (m) | Surface Salinity (ppt) | -0.0857 | 0.7353 |
| NH3&NH4+ (ug/L) | Bottom Temp (ºC) | -0.0864 | 0.7333 |
| nifH/16S | Redox | -0.0868 | 0.7321 |
| Depth (m) | Distance from E16 (km) | -0.0898 | 0.7231 |
| ng/µL_extracted | NO2-+NO3- (ug/L) | -0.0898 | 0.7231 |
| NH3&NH4+ (ug/L) | Surface Temp (ºC) | -0.0914 | 0.7182 |
| Sand (%) | Secchi (cm) | -0.0922 | 0.7159 |
| nifH/16S | DEA +N (ppmv h-1) | -0.0939 | 0.7109 |
| nirS copy number | DRP (ug/L) | -0.1001 | 0.6927 |
| TP (ug/L) | Surface Temp (ºC) | -0.1008 | 0.6907 |
| nirS/16S | Bottom Temp (ºC) | -0.1009 | 0.6902 |
| nosZII/16S | TP (ug/L) | -0.1022 | 0.6867 |
| NH3&NH4+ (ug/L) | Redox | -0.1023 | 0.6863 |
| Distance from E2-E3 (m) | Secchi (cm) | -0.1047 | 0.6794 |
| Secchi (cm) | Surface Salinity (ppt) | -0.1058 | 0.6762 |
| DEA +C+N (ppmv h-1) | Bottom Salinity (ppt) | -0.1063 | 0.6746 |
| DRP (ug/L) | Distance from E16 (km) | -0.1084 | 0.6687 |
| Silt (%) | Distance from E16 (km) | -0.1104 | 0.6627 |
| Porosity (%) | Distance from E16 (km) | -0.1125 | 0.6568 |
| ng/µL_extracted | Surface Salinity (ppt) | -0.1177 | 0.6418 |
| ng/µL_extracted | DEA +C+N (ppmv h-1) | -0.1207 | 0.6332 |
| nirS copy number | NO2-+NO3- (ug/L) | -0.1249 | 0.6215 |
| Shannon | Distance from E16 (km) | -0.1249 | 0.6215 |
| Organic Matter (%) | Distance from E16 (km) | -0.1269 | 0.6157 |
| nosZII copy number | NO2-+NO3- (ug/L) | -0.1269 | 0.6157 |
| DEA +C+N (ppmv h-1) | Silt (%) | -0.1269 | 0.6157 |
| nifH copy number | TN (ug/L) | -0.1311 | 0.6042 |
| TN (ug/L) | Distance from E16 (km) | -0.1331 | 0.5985 |
| Porosity (%) | DEA +N (ppmv h-1) | -0.1331 | 0.5985 |
| Richness | DRP (ug/L) | -0.1342 | 0.5954 |
| TP (ug/L) | Distance from E16 (km) | -0.1352 | 0.5927 |
| nifH copy number | DRP (ug/L) | -0.1373 | 0.5871 |
| nosZI/16S | Distance from E16 (km) | -0.1373 | 0.5871 |
| TN (ug/L) | Depth (m) | -0.1393 | 0.5814 |
| DEA +N (ppmv h-1) | Clay (%) | -0.1414 | 0.5758 |
| DEA +C+N (ppmv h-1) | DRP (ug/L) | -0.1476 | 0.559 |
| TN (ug/L) | Surface Temp (ºC) | -0.1517 | 0.5479 |
| NH3&NH4+ (ug/L) | NO2-+NO3- (ug/L) | -0.1517 | 0.5479 |
| DEA +N (ppmv h-1) | Organic Matter (%) | -0.1517 | 0.5479 |
| ng/µL_extracted | Shannon | -0.1517 | 0.5479 |
| Clay (%) | DRP (ug/L) | -0.1538 | 0.5424 |
| DRP (ug/L) | Surface Salinity (ppt) | -0.1569 | 0.534 |
| ng/µL_extracted | TP (ug/L) | -0.1579 | 0.5315 |
| Redox | Bottom Salinity (ppt) | -0.1612 | 0.5229 |
| nirS/16S | TP (ug/L) | -0.162 | 0.5207 |
| nosZI/16S | Surface Temp (ºC) | -0.1694 | 0.5017 |
| ng/µL_extracted | Redox | -0.1694 | 0.5015 |
| DEA +C+N (ppmv h-1) | Bottom Temp (ºC) | -0.1696 | 0.501 |
| nirS/16S | Silt (%) | -0.1703 | 0.4993 |
| nosZII copy number | DRP (ug/L) | -0.1723 | 0.4941 |
| 16S copy number | DRP (ug/L) | -0.1723 | 0.4941 |
| Distance from E2-E3 (m) | DEA +N (ppmv h-1) | -0.1723 | 0.4941 |
| nirS/16S | Secchi (cm) | -0.1731 | 0.4923 |
| nosZI copy number | TN (ug/L) | -0.1744 | 0.4888 |
| nirS/16S | Distance from E16 (km) | -0.1765 | 0.4836 |
| Surface Salinity (ppt) | Distance from E16 (km) | -0.1786 | 0.4782 |
| ng/µL_extracted | Depth (m) | -0.1847 | 0.4631 |
| Clay (%) | TN (ug/L) | -0.195 | 0.438 |
| nifH copy number | NO2-+NO3- (ug/L) | -0.195 | 0.438 |
| Depth (m) | Redox | -0.1983 | 0.4301 |
| Porosity (%) | DRP (ug/L) | -0.1992 | 0.4282 |
| DRP (ug/L) | Organic Matter (%) | -0.2033 | 0.4184 |
| nirS/16S | Distance from E2-E3 (m) | -0.2033 | 0.4184 |
| nosZI copy number | DRP (ug/L) | -0.2054 | 0.4136 |
| DEA +C+N (ppmv h-1) | Surface Temp (ºC) | -0.2088 | 0.4056 |
| Richness | TN (ug/L) | -0.2127 | 0.3968 |
| Secchi (cm) | Surface Temp (ºC) | -0.2134 | 0.3953 |
| 16S copy number | TN (ug/L) | -0.2157 | 0.39 |
| ng/µL_extracted | NH3&NH4+ (ug/L) | -0.2178 | 0.3854 |
| nirS copy number | Distance from E16 (km) | -0.2198 | 0.3808 |
| nirS/16S | nosZII copy number | -0.2198 | 0.3808 |
| Richness | nosZI copy number | -0.223 | 0.3737 |
| Organic Matter (%) | Redox | -0.2231 | 0.3734 |
| nifH copy number | Redox | -0.2242 | 0.3712 |
| TP (ug/L) | Bottom Temp (ºC) | -0.2279 | 0.3631 |
| DEA +N (ppmv h-1) | Surface Salinity (ppt) | -0.2334 | 0.3514 |
| nosZII copy number | TN (ug/L) | -0.2343 | 0.3495 |
| DEA +N (ppmv h-1) | TP (ug/L) | -0.2343 | 0.3495 |
| ng/µL_extracted | Silt (%) | -0.2363 | 0.3451 |
| Silt (%) | Redox | -0.2366 | 0.3446 |
| nosZI/16S | Bottom Temp (ºC) | -0.2529 | 0.3113 |
| nosZI copy number | Secchi (cm) | -0.2549 | 0.3073 |
| nirS/16S | nifH copy number | -0.257 | 0.3033 |
| TN (ug/L) | Bottom Salinity (ppt) | -0.2632 | 0.2914 |
| nirS/16S | NH3&NH4+ (ug/L) | -0.2632 | 0.2914 |
| DEA +N (ppmv h-1) | Depth (m) | -0.2652 | 0.2875 |
| nirS/16S | Bottom Salinity (ppt) | -0.2673 | 0.2836 |
| nirS/16S | Clay (%) | -0.2714 | 0.276 |
| Porosity (%) | TN (ug/L) | -0.2735 | 0.2722 |
| Porosity (%) | Redox | -0.2748 | 0.2698 |
| nirS/16S | 16S copy number | -0.2755 | 0.2684 |
| 16S copy number | NO2-+NO3- (ug/L) | -0.2776 | 0.2647 |
| nosZII/16S | DRP (ug/L) | -0.2776 | 0.2647 |
| Silt (%) | DRP (ug/L) | -0.2838 | 0.2538 |
| nosZI copy number | nifH copy number | -0.2859 | 0.2502 |
| DEA +N (ppmv h-1) | Bottom Salinity (ppt) | -0.2859 | 0.2502 |
| NO2-+NO3- (ug/L) | Depth (m) | -0.2879 | 0.2466 |
| nosZII/16S | nosZI/16S | -0.2879 | 0.2466 |
| nirS/16S | Depth (m) | -0.2921 | 0.2396 |
| nirS/16S | Organic Matter (%) | -0.2921 | 0.2396 |
| TN (ug/L) | Organic Matter (%) | -0.2962 | 0.2327 |
| Sand (%) | NH3&NH4+ (ug/L) | -0.2962 | 0.2327 |
| nirS/16S | Porosity (%) | -0.2962 | 0.2327 |
| Clay (%) | Redox | -0.2996 | 0.2271 |
| nosZI/16S | Shannon | -0.3044 | 0.2193 |
| 16S copy number | Redox | -0.3048 | 0.2188 |
| ng/µL_extracted | Bottom Salinity (ppt) | -0.3086 | 0.2128 |
| Sand (%) | TP (ug/L) | -0.3127 | 0.2065 |
| Shannon | Redox | -0.3182 | 0.1982 |
| DRP (ug/L) | Bottom Salinity (ppt) | -0.3189 | 0.1971 |
| DEA +C+N (ppmv h-1) | Clay (%) | -0.3189 | 0.1971 |
| nosZI copy number | Surface Salinity (ppt) | -0.3191 | 0.1969 |
| ng/µL_extracted | Richness | -0.3201 | 0.1954 |
| nirS/16S | Surface Salinity (ppt) | -0.3201 | 0.1954 |
| Porosity (%) | DEA +C+N (ppmv h-1) | -0.3209 | 0.1941 |
| nosZII/16S | ng/µL_extracted | -0.3209 | 0.1941 |
| Porosity (%) | NO2-+NO3- (ug/L) | -0.3251 | 0.1881 |
| Sand (%) | Bottom Temp (ºC) | -0.3257 | 0.1872 |
| NO2-+NO3- (ug/L) | Organic Matter (%) | -0.3292 | 0.1822 |
| nosZI/16S | TP (ug/L) | -0.3313 | 0.1793 |
| nifH/16S | ng/µL_extracted | -0.3333 | 0.1765 |
| nifH/16S | Distance from E2-E3 (m) | -0.3354 | 0.1736 |
| Redox | Distance from E16 (km) | -0.3357 | 0.1732 |
| ng/µL_extracted | Secchi (cm) | -0.3389 | 0.169 |
| DEA +C+N (ppmv h-1) | Organic Matter (%) | -0.3416 | 0.1653 |
| nosZI/16S | Secchi (cm) | -0.3472 | 0.1581 |
| nosZI copy number | TP (ug/L) | -0.3478 | 0.1573 |
| nirS copy number | Redox | -0.3523 | 0.1517 |
| DEA +N (ppmv h-1) | Redox | -0.3523 | 0.1517 |
| Sand (%) | Surface Temp (ºC) | -0.3553 | 0.1479 |
| nosZII copy number | Redox | -0.3657 | 0.1356 |
| nosZI copy number | Distance from E16 (km) | -0.3684 | 0.1325 |
| Distance from E2-E3 (m) | NH3&NH4+ (ug/L) | -0.3684 | 0.1325 |
| nosZII copy number | nosZI copy number | -0.3705 | 0.1302 |
| nosZI copy number | NH3&NH4+ (ug/L) | -0.3725 | 0.1279 |
| Distance from E2-E3 (m) | TP (ug/L) | -0.3746 | 0.1256 |
| nifH/16S | nosZI copy number | -0.3746 | 0.1256 |
| 16S copy number | nosZI copy number | -0.3767 | 0.1234 |
| DEA +C+N (ppmv h-1) | Surface Salinity (ppt) | -0.3779 | 0.1221 |
| nosZII/16S | Sand (%) | -0.3787 | 0.1212 |
| nosZI copy number | Organic Matter (%) | -0.3911 | 0.1085 |
| nosZII/16S | Redox | -0.3915 | 0.1081 |
| NO2-+NO3- (ug/L) | Bottom Salinity (ppt) | -0.3932 | 0.1065 |
| nosZI copy number | Silt (%) | -0.3973 | 0.1025 |
| nirS/16S | Redox | -0.405 | 0.0955 |
| Clay (%) | NO2-+NO3- (ug/L) | -0.4056 | 0.095 |
| Porosity (%) | nosZI copy number | -0.4056 | 0.095 |
| nosZI copy number | Bottom Salinity (ppt) | -0.4097 | 0.0913 |
| DEA +C+N (ppmv h-1) | Depth (m) | -0.4118 | 0.0895 |
| ng/µL_extracted | nifH copy number | -0.42 | 0.0827 |
| nosZI/16S | Organic Matter (%) | -0.42 | 0.0827 |
| Richness | Redox | -0.4253 | 0.0785 |
| TN (ug/L) | Bottom Temp (ºC) | -0.4339 | 0.072 |
| Silt (%) | NO2-+NO3- (ug/L) | -0.4345 | 0.0716 |
| nosZII/16S | TN (ug/L) | -0.4427 | 0.0658 |
| ng/µL_extracted | 16S copy number | -0.4613 | 0.054 |
| DRP (ug/L) | Surface Temp (ºC) | -0.4748 | 0.0465 |
| DEA +C+N (ppmv h-1) | Redox | -0.4762 | 0.0457 |
| NO2-+NO3- (ug/L) | Bottom Temp (ºC) | -0.4797 | 0.0439 |
| nosZI/16S | nifH/16S | -0.4943 | 0.037 |
| nosZI copy number | Clay (%) | -0.4985 | 0.0353 |
| nosZI/16S | Porosity (%) | -0.5067 | 0.0319 |
| nirS copy number | Sand (%) | -0.5212 | 0.0266 |
| nosZI/16S | nirS copy number | -0.5232 | 0.0259 |
| nosZI/16S | NH3&NH4+ (ug/L) | -0.5253 | 0.0252 |
| ng/µL_extracted | nosZII copy number | -0.5459 | 0.0191 |
| nosZI copy number | Depth (m) | -0.5501 | 0.018 |
| DRP (ug/L) | Bottom Temp (ºC) | -0.5526 | 0.0174 |
| nosZI/16S | Surface Salinity (ppt) | -0.5772 | 0.0121 |
| NO2-+NO3- (ug/L) | Surface Temp (ºC) | -0.5839 | 0.011 |
| ng/µL_extracted | nirS copy number | -0.5851 | 0.0107 |
| Shannon | Sand (%) | -0.6244 | 0.0056 |
| nosZI/16S | Richness | -0.6278 | 0.0053 |
| nifH/16S | Sand (%) | -0.6326 | 0.0048 |
| Distance from E2-E3 (m) | DRP (ug/L) | -0.6471 | 0.0037 |
| nosZI/16S | Clay (%) | -0.6533 | 0.0033 |
| nosZI/16S | Silt (%) | -0.6533 | 0.0033 |
| nosZI/16S | Bottom Salinity (ppt) | -0.6718 | 0.0023 |
| Distance from E2-E3 (m) | TN (ug/L) | -0.6842 | 0.0017 |
| Sand (%) | Surface Salinity (ppt) | -0.697 | 0.0013 |
| Sand (%) | Bottom Salinity (ppt) | -0.7069 | 0.001 |
| nosZI/16S | Depth (m) | -0.7337 | 0.0005 |
| Richness | Sand (%) | -0.7424 | 0.0004 |
| NO2-+NO3- (ug/L) | Surface Salinity (ppt) | -0.7548 | 0.0003 |
| nosZII copy number | Sand (%) | -0.7936 | <.0001 |
| 16S copy number | Sand (%) | -0.8287 | <.0001 |
| Sand (%) | Organic Matter (%) | -0.8308 | <.0001 |
| nifH copy number | Sand (%) | -0.8308 | <.0001 |
| Porosity (%) | Sand (%) | -0.8638 | <.0001 |
| nosZI/16S | nifH copy number | -0.8658 | <.0001 |
| Sand (%) | Depth (m) | -0.8762 | <.0001 |
| nosZI/16S | nosZII copy number | -0.9051 | <.0001 |
| nosZI/16S | 16S copy number | -0.9319 | <.0001 |
| Sand (%) | Clay (%) | -0.9463 | <.0001 |
| Sand (%) | Silt (%) | -0.9794 | <.0001 |

**Table S3** Spearman’s rank correlation between normalized normalised *nirS* values (nirS%) and OTUs identified by 16S sequencing.

| Variable | by Variable | Spearman ρ | Prob>\|ρ\| |
| --- | --- | --- | --- |
| nirS% | Bacteria, Proteobacteria, Alphaproteobacteria, Rhizobiales, Rhodobiaceae, Rhodobium, uncultured bacterium | 0.6593 | <.0001 |
| nirS% | Bacteria, Proteobacteria, Betaproteobacteria, Burkholderiales, Alcaligenaceae, uncultured, NA | 0.6188 | <.0001 |
| nirS% | Bacteria, Actinobacteria, Thermoleophilia, Solirubrobacterales, 480-2, uncultured bacterium, uncultured bacterium | 0.5552 | <.0001 |
| nirS% | Bacteria, Gemmatimonadetes, Gemmatimonadetes, Gemmatimonadales, Gemmatimonadaceae, uncultured, NA | 0.5516 | <.0001 |
| nirS% | Bacteria, Actinobacteria, Thermoleophilia, Solirubrobacterales, 480-2, NA, NA | 0.5484 | <.0001 |
| nirS% | Bacteria, Proteobacteria, Deltaproteobacteria, Desulfobacterales, Desulfobulbaceae, Desulfobulbus, uncultured delta proteobacterium | 0.5178 | <.0001 |
| nirS% | Bacteria, Proteobacteria, Gammaproteobacteria, Chromatiales, Ectothiorhodospiraceae, Acidiferrobacter, uncultured gamma proteobacterium | 0.4512 | <.0001 |
| nirS% | Bacteria, Proteobacteria, Deltaproteobacteria, Myxococcales, Sandaracinaceae, uncultured delta proteobacterium, uncultured delta proteobacterium | 0.3592 | 0.0019 |
| nirS% | Bacteria, Proteobacteria, Deltaproteobacteria, Myxococcales, Sandaracinaceae, uncultured delta proteobacterium, uncultured delta proteobacterium 3 | 0.3426 | 0.0032 |
| nirS% | Bacteria, Proteobacteria, Gammaproteobacteria, Xanthomonadales, uncultured, uncultured gamma proteobacterium, uncultured gamma proteobacterium | 0.3295 | 0.0047 |
| nirS% | Bacteria, Proteobacteria, Alphaproteobacteria, Sphingomonadales, Sphingomonadaceae, Sphingomonas, uncultured bacterium | 0.2743 | 0.0197 |
| nirS% | Bacteria, Actinobacteria, Acidimicrobiia, Acidimicrobiales, OM1 clade, uncultured bacterium, uncultured bacterium 2 | 0.2683 | 0.0227 |
| nirS% | Bacteria, Firmicutes, Bacilli, Bacillales, Paenibacillaceae, Paenibacillus, NA | 0.2533 | 0.0318 |
| nirS% | Bacteria, Proteobacteria, Gammaproteobacteria, Xanthomonadales, JTB255 marine benthic group, uncultured bacterium, uncultured bacterium | 0.241 | 0.0414 |
| nirS% | Bacteria, Proteobacteria, Gammaproteobacteria, Pseudomonadales, Pseudomonadaceae, Pseudomonas, uncultured bacterium | 0.2408 | 0.0416 |
| nirS% | Bacteria, Proteobacteria, Alphaproteobacteria, Rhodobacterales, Rhodobacteraceae, uncultured, uncultured bacterium | 0.2233 | 0.0594 |
| nirS% | Bacteria, Actinobacteria, Acidimicrobiia, Acidimicrobiales, OM1 clade,NA,NA | 0.2169 | 0.0673 |
| nirS% | Bacteria, Actinobacteria, Acidimicrobiia, Acidimicrobiales, OM1 clade, uncultured bacterium, uncultured bacterium 3 | 0.1912 | 0.1077 |
| nirS% | Bacteria, Cyanobacteria, Chloroplast, uncultured bacterium, uncultured bacterium, uncultured bacterium, uncultured bacterium | 0.1846 | 0.1205 |
| nirS% | Bacteria, Proteobacteria, Gammaproteobacteria, Xanthomonadales, JTB255 marine benthic group, uncultured bacterium, uncultured bacterium 3 | 0.0883 | 0.4608 |
| nirS% | Bacteria, Proteobacteria, Gammaproteobacteria, Xanthomonadales, JTB255 marine benthic group, uncultured bacterium, uncultured bacterium 2 | 0.0773 | 0.5185 |
| nirS% | Bacteria, Proteobacteria, Deltaproteobacteria, Desulfobacterales, Desulfobacteraceae, Sva0081 sediment group, uncultured delta proteobacterium | 0.0528 | 0.6594 |
| nirS% | Bacteria, Cyanobacteria, Chloroplast, uncultured phototrophic eukaryote, uncultured phototrophic eukaryote, uncultured phototrophic eukaryote, uncultured phototrophic eukaryote | 0.0448 | 0.7085 |
| nirS% | Bacteria, Actinobacteria, Acidimicrobiia, Acidimicrobiales, uncultured, uncultured bacterium, uncultured bacterium | 0.0419 | 0.7267 |
| nirS% | Bacteria, Cyanobacteria, Cyanobacteria, SubsectionI, FamilyI, Synechococcus, uncultured bacterium | 0.0395 | 0.7416 |
| nirS% | Bacteria, Proteobacteria, Betaproteobacteria, Burkholderiales, Comamonadaceae, NA, NA | 0.0301 | 0.802 |
| nirS% | Bacteria, Proteobacteria, Deltaproteobacteria, Desulfobacterales, Desulfobacteraceae, NA, NA | -0.015 | 0.9007 |
| nirS% | Bacteria, Proteobacteria, Gammaproteobacteria, Order Incertae Sedis, Family Incertae Sedis, Marinicella, NA | -0.0496 | 0.6789 |
| nirS% | Bacteria, Actinobacteria, Acidimicrobiia, Acidimicrobiales, OM1 clade, uncultured bacterium, uncultured bacterium | -0.0984 | 0.411 |
| nirS% | Bacteria, Bacteroidetes, Flavobacteriia, Flavobacteriales, NS9 marine group, uncultured bacterium, uncultured bacterium | -0.1398 | 0.2413 |
| nirS% | Bacteria, Cyanobacteria, Cyanobacteria, SubsectionI, FamilyI, Synechococcus, uncultured bacterium 2 | -0.1546 | 0.1946 |
| nirS% | Bacteria, Proteobacteria, Deltaproteobacteria, Desulfuromonadales, Sva1033, uncultured bacterium, uncultured bacterium | -0.1569 | 0.1882 |
| nirS% | Bacteria, Proteobacteria, Deltaproteobacteria, Myxococcales, Sandaracinaceae, uncultured delta proteobacterium, uncultured delta proteobacterium 2 | -0.2087 | 0.0786 |
| nirS% | Bacteria, Acidobacteria, Holophagae, Subgroup 23, uncultured bacterium, uncultured bacterium, uncultured bacterium | -0.2165 | 0.0678 |
| nirS% | Bacteria, Bacteroidetes, VC2.1 Bac22, NA, NA, NA, NA | -0.2185 | 0.0651 |
| nirS% | Bacteria, Proteobacteria, Alphaproteobacteria, Rhodospirillales, Rhodospirillaceae, Defluviicoccus, NA | -0.2252 | 0.0572 |
| nirS% | Bacteria, Chlorobi, Ignavibacteria, Ignavibacteriales, PHOS-HE36, uncultured soil bacterium, uncultured soil bacterium | -0.2447 | 0.0383 |
| nirS% | Bacteria, Bacteroidetes, SB-5, uncultured organism, uncultured organism, uncultured organism, uncultured organism | -0.2725 | 0.0206 |
| nirS% | Bacteria, Proteobacteria, Deltaproteobacteria, Myxococcales, Sandaracinaceae, NA, NA | -0.2902 | 0.0134 |
| nirS% | Bacteria, Bacteroidetes, Flavobacteriia, Flavobacteriales, Flavobacteriaceae, Lutibacter, NA | -0.2938 | 0.0122 |
| nirS% | Bacteria, Cyanobacteria, Chloroplast, uncultured bacterium, uncultured bacterium, uncultured bacterium, uncultured bacterium 2 | -0.353 | 0.0024 |
| nirS% | Bacteria, Proteobacteria, Gammaproteobacteria, Alteromonadales, Alteromonadaceae, NA, NA | -0.3681 | 0.0015 |
| nirS% | Bacteria, Cyanobacteria, Cyanobacteria, SubsectionI, FamilyI, Synechococcus, NA | -0.523 | <.0001 |
| nirS% | Bacteria, Proteobacteria, Deltaproteobacteria, Desulfobacterales, Desulfobulbaceae, uncultured, NA | -0.5746 | <.0001 |

**Table S4** Copy numbers per gram of sediment for all functional genes.

| **Site** | ***nifH*** | | ***nirS*** | | ***nosZI*** | | ***nosZII*** | |
| --- | --- | --- | --- | --- | --- | --- | --- | --- |
|  | **Mean** | **Std Err** | **Mean** | **Std Err** | **Mean** | **Std Err** | **Mean** | **Std Err** |
| **E1** | 2.07E+08 | 1.26E+07 | 1.99E+08 | 2.94E+07 | 2.47E+08 | 9.16E+07 | 1.22E+08 | 2.95E+07 |
| **E10** | 1.12E+09 | 1.57E+08 | 1.73E+08 | 1.54E+07 | 1.35E+08 | 2.70E+07 | 5.74E+08 | 7.13E+07 |
| **E11** | 1.09E+09 | 8.75E+07 | 1.71E+08 | 1.76E+07 | 8.87E+07 | 1.03E+07 | 5.53E+08 | 7.99E+07 |
| **E12** | 6.39E+08 | 1.48E+08 | 3.44E+08 | 4.30E+07 | 3.21E+08 | 1.03E+08 | 3.06E+08 | 6.49E+07 |
| **E13** | 7.40E+08 | 5.01E+07 | 3.77E+08 | 1.48E+07 | 2.73E+08 | 3.26E+07 | 4.19E+08 | 2.43E+07 |
| **E14** | 3.50E+08 | 3.34E+07 | 2.94E+08 | 2.12E+07 | 2.29E+08 | 3.96E+07 | 2.84E+08 | 2.64E+07 |
| **E15** | 1.83E+08 | 8.33E+07 | 1.15E+08 | 4.23E+07 | 3.11E+08 | 1.11E+08 | 8.69E+07 | 2.29E+07 |
| **E16** | 3.53E+08 | 2.13E+07 | 1.49E+08 | 1.02E+07 | 2.54E+08 | 3.98E+07 | 1.33E+08 | 5.55E+06 |
| **E17** | 3.08E+08 | 1.10E+07 | 2.09E+08 | 1.71E+06 | 1.36E+08 | 1.07E+07 | 2.27E+08 | 9.70E+06 |
| **E18** | 5.87E+08 | 5.31E+07 | 3.75E+08 | 3.56E+07 | 1.75E+08 | 2.24E+07 | 3.29E+08 | 3.81E+07 |
| **E2** | 8.57E+07 | 1.04E+07 | 3.53E+07 | 3.33E+06 | 1.09E+08 | 2.34E+07 | 2.09E+07 | 3.57E+06 |
| **E3** | 2.55E+08 | 6.85E+07 | 1.71E+08 | 3.34E+07 | 2.10E+08 | 4.70E+07 | 1.02E+08 | 1.88E+07 |
| **E4** | 4.46E+08 | 3.41E+07 | 2.06E+08 | 2.03E+07 | 4.97E+07 | 5.72E+06 | 2.37E+08 | 1.02E+07 |
| **E5** | 5.77E+08 | 6.34E+07 | 1.66E+08 | 6.54E+06 | 6.10E+07 | 5.89E+06 | 2.92E+08 | 2.89E+07 |
| **E6** | 1.69E+08 | 4.52E+07 | 8.95E+07 | 1.67E+07 | 1.72E+08 | 4.61E+07 | 6.04E+07 | 1.50E+07 |
| **E7** | 1.70E+08 | 6.40E+07 | 1.24E+08 | 4.29E+07 | 3.28E+08 | 8.47E+07 | 1.32E+08 | 2.32E+07 |
| **E8** | 2.07E+08 | 3.99E+07 | 1.81E+08 | 3.33E+07 | 8.78E+07 | 1.60E+07 | 2.40E+08 | 3.55E+07 |
| **E9** | 9.05E+08 | 7.21E+07 | 1.62E+08 | 1.07E+07 | 1.00E+08 | 1.08E+07 | 3.96E+08 | 2.31E+07 |
|  |  |  |  |  |  |  |  |  |
| **Min** | 8.57E+07 |  | 3.53E+07 |  | 4.97E+07 |  | 2.09E+07 |  |
| **Max** | 1.12E+09 |  | 3.77E+08 |  | 3.28E+08 |  | 5.74E+08 |  |
| **Median** | 3.52E+08 |  | 1.72E+08 |  | 1.73E+08 |  | 2.38E+08 |  |
